# The mammary gland-specific marsupial *ELP* and eutherian *CTI* share a common ancestral gene

**DOI:** 10.1186/1471-2148-12-80

**Published:** 2012-06-08

**Authors:** Elizabeth A Pharo, Alison A De Leo, Marilyn B Renfree, Peter C Thomson, Christophe M Lefèvre, Kevin R Nicholas

**Affiliations:** 1 Department of Zoology, The University of Melbourne, Melbourne, Victoria, 3010, Australia; 2 Cooperative Research Centre for Innovative Dairy Products; 3 ARC Centre of Excellence for Kangaroo Genomics; 4 Faculty of Veterinary Science, The University of Sydney, Sydney, NSW, 2006, Australia; 5 Institute for Technology Research and Innovation, Deakin University, Geelong, Victoria, 3214, Australia

## Abstract

**Background:**

The marsupial *early lactation protein* (*ELP*) gene is expressed in the mammary gland and the protein is secreted into milk during early lactation (Phase 2A). Mature ELP shares approximately 55.4% similarity with the colostrum-specific bovine colostrum trypsin inhibitor (CTI) protein. Although ELP and CTI both have a single bovine pancreatic trypsin inhibitor (BPTI)-Kunitz domain and are secreted only during the early lactation phases, their evolutionary history is yet to be investigated.

**Results:**

Tammar *ELP* was isolated from a genomic library and the fat-tailed dunnart and Southern koala *ELP* genes cloned from genomic DNA. The tammar *ELP* gene was expressed only in the mammary gland during late pregnancy (Phase 1) and early lactation (Phase 2A). The opossum and fat-tailed dunnart *ELP* and cow *CTI* transcripts were cloned from RNA isolated from the mammary gland and dog *CTI* from cells in colostrum. The putative mature ELP and CTI peptides shared 44.6%-62.2% similarity. *In silico* analyses identified the *ELP* and *CTI* genes in the other species examined and provided compelling evidence that they evolved from a common ancestral gene. In addition, whilst the eutherian *CTI* gene was conserved in the Laurasiatherian orders Carnivora and Cetartiodactyla, it had become a pseudogene in others. These data suggest that bovine *CTI* may be the ancestral gene of the Artiodactyla-specific, rapidly evolving chromosome 13 *pancreatic trypsin inhibitor* (*PTI*), *spleen trypsin inhibitor* (*STI*) and the five placenta-specific *trophoblast Kunitz domain protein* (*TKDP1-5*) genes.

**Conclusions:**

Marsupial *ELP* and eutherian *CTI* evolved from an ancestral therian mammal gene before the divergence of marsupials and eutherians between 130 and 160 million years ago*.* The retention of the *ELP* gene in marsupials suggests that this early lactation-specific milk protein may have an important role in the immunologically naïve young of these species.

## Background

Marsupials and eutherians diverged between 130 and 160 million years ago [1-3] and evolved very different reproductive strategies [4-6]. Marsupials have an ultra-short gestation ranging from 10.7 days for the stripe-faced dunnart (*Smithopsis macroura*) [7] to 38 days for the long-nosed potoroo (*Potorous tridactylus*) [8] and deliver an altricial young [5].

Organogenesis is completed after birth supported by a long and physiologically complex lactation, during which there is an increase in maternal mammary gland size and milk production, and there are dramatic changes in milk composition [5,9-13]. In contrast, eutherians have a long pregnancy during which maternal investment is high [14,15]. During eutherian lactation, milk composition remains relatively constant apart from the initial production of colostrum 24–36 hr postpartum (pp) [16].

The tammar wallaby (*Macropus eugenii*) has a 26.5-day pregnancy after embryonic diapause [17]. After giving birth, the tammar produces milk for ~300 days until the young is weaned. Phase 1 of lactation is comprised of mammary development during pregnancy and lactogenesis around parturition. At birth, the altricial young (~400 mg) attaches to one of the four teats [5,9,13,18]. Lactation proceeds only in the sucked gland, whilst the remaining three glands regress [5,9]. The young remains permanently attached to the teat from the day of birth until day 100 pp (Phase 2A) followed by detachment from the teat and a period of intermittent sucking while confined in the pouch between days 100–200 pp (Phase 2B) [5,13,18]. The final phase is from day 200 to at least day 300 when the young suckles variably and begins to graze as well as maintaining a milk intake (Phase 3) [18]. These phases are highly correlated with changes in milk composition and mammary gland gene expression [10,13,19]. Milk protein genes such as *α-lactalbuminβ-lactoglobulin* (*LGB*), *α-caseinβ-casein* and *κ-casein* are induced at parturition and expressed throughout lactation, whilst others are expressed and secreted in a phase-specific manner [13]. *Early lactation protein* (*ELP*) is expressed during Phase 2A only [13,20,21], *whey acidic protein* (*WAP*) is Phase 2B-specific [22] and *late lactation protein A* and *B* are characteristic to late Phase 2B/Phase 3 and Phase 3 respectively [23,24].

The *ELP* gene was first identified in an Australian marsupial, the brushtail possum (*Trichosurus vulpecula*) [25]. *ELP* encodes a small precursor protein with a single bovine pancreatic trypsin inhibitor (BPTI)-Kunitz domain characteristic to serine protease inhibitors. ELP is secreted in milk in multiple isoforms, which include an ~8 kDa peptide and a heavily N-glycosylated protein (~16 kDa) [25]. *ELP* was later identified in the tammar [13,20,21,26], the stripe-faced and fat-tailed dunnarts (*Sminthopsis macroura* and *Sminthopsis crassicaudata* respectively) and the South American grey short-tailed opossum (*Monodelphis domestica*) [27] (Refer to Additional file 1: Table S1 for the species in which the putative functional *ELP*/*CTI* gene, transcript and protein have been identified). Marsupial *ELP* expression is limited to the early phase of lactation [13,20,21,27,28] at the time the mother produces milk for an immunologically naïve young [29,30]. During this period, the tammar young is permanently attached to the teat and protected by humoral (passive) immunity acquired from its mother’s milk and its own innate immunity [18,30].

Whilst an *ELP* orthologue is yet to be identified in eutherians, tammar and possum ELP share ~37% similarity with bovine colostrum trypsin inhibitor (CTI) [20,25]. CTI was discovered by chance in bovine colostrum over 60 years ago [31]. Putative CTI proteins with trypsin inhibitor activity were subsequently isolated from colostrum of the pig [32], cat, sheep, goat, dog, reindeer, ferret and Blue fox [33], but were not found in equine colostrum [34]. These glycosylated proteins inhibited serine endopeptidases such as trypsin, pepsin and chymotrypsin [31,32,35]. However, of these putative CTI proteins, only bovine CTI has been sequenced (Additional file 1: Table S1) and found to contain a Kunitz domain which generally indicates serine protease inhibitor activity (see below) [36]. Laskowski and Laskowski [31] hypothesised that bovine CTI protected immunoglobulins against proteolysis during the crucial period of immunoglobulin transfer from cow to calf via colostrum. However, its function is yet to be determined. Although CTI and ELP are expressed in early milk, bovine CTI secretion is brief (~1-2 days) [31,37], but marsupial ELP expression is prolonged (up to 100 days pp) [20,21,25,28]. However, their secretion in milk is correlated with the period of immuno-incompetence in the young [29,31].

The Kunitz domain was thought to have evolved over 500 million years ago [38] and is now ubiquitous in mammals, reptiles, birds, plants, insects, nematodes, venoms from snakes, spiders, cone snails and sea anemones and in viruses and bacteria [39-42]. The archetypal protein of the Kunitz domain and the BPTI-Kunitz family I2, clan IB of serine endopeptidase inhibitors in the MEROPS database [43,44] is the much studied bovine pancreatic trypsin inhibitor, also known as aprotinin (reviewed in [45]). The Kunitz domain is characterised by six conserved cysteine residues which form three disulphide bonds, producing a compact, globular protein of α + β folds [43,46,47]. Serine endopeptidase inhibition occurs through the binding of the P_1_ reactive site residue within the ‘binding loop’ of the Kunitz domain to a serine residue within the catalytic cleft of the protease [47,48]. This is a reversible, tight-binding, 1:1 interaction [44,48]. Furthermore, the Kunitz domain P_1_ residue determines protease-specificity [39,47].

Since its evolution, the Kunitz domain has been incorporated into many different genes [43,44]. In general, each domain is encoded by a single exon [43,49]. Some genes encode proteins with a single Kunitz domain, e.g. *ELPCTIPTIspleen trypsin inhibitor* (*STI*), the five *trophoblast Kunitz domain protein* genes (*TKDP1-5*) and *serine protease inhibitor Kunitz-type-3* (*SPINT3*) and *SPINT4*. These genes, apart from the TKDPs, have 3 exons. The first exon encodes the signal- and pro-peptide, the second, a single Kunitz domain and the third, a short C-terminus. However, the TKDPs have a variable number of unique N domains inserted between the signal peptide and the Kunitz domain-encoding exon [50,51]. Genes that encode multiple Kunitz domains include: *hepatocyte growth factor activator inhibitor 1* and *2*, also known as *SPINT1* and *SPINT2* respectively (two domains), *tissue factor pathway inhibitor 1* and *2* (three domains); with up to 12 domains in the Ac-KPI-1 I nematode (*Ancylostoma caninum*) protein [38,43,44]. In addition, the Kunitz domain has been integrated into multi-domain proteins, some of which include: the collagen α3(VI), α1(VII) and α1(XXVIII) chains, WFDC6 and WFDC8, amyloid beta A4 protein, α1-microglobulin/bikunin precursor (AMBP), SPINLW1 [serine peptidase inhibitor-like, with Kunitz and WAP domains 1 (eppin)] and the WAP, follistatin/kazal, immunoglobulin, Kunitz and netrin domain containing (WFIKKN)1 and 2 proteins [39]. Furthermore, each domain within a multi-Kunitz domain protein, may exhibit different protease activity, such as for the three tandemly repeated domains within both tissue factor pathway inhibitor 1 and 2 [43,44,52].

The early lactation/colostrum-specific expression of *ELP*/*CTI* suggests these Kunitz domain-encoding genes may play an important role in the neonate. The sequencing of the tammar genome [53], in addition to the availability of numerous vertebrate genomes including one other marsupial, the opossum, a monotreme, the platypus, many eutherians, birds (chicken, Zebra finch), fish (Zebrafish, Japanese medaka, Three-spine stickleback, Tiger and Green spotted puffers), amphibian (African clawed frog) and reptile (Green anole lizard), provides an invaluable resource with which to investigate the evolution of these genes. We used a comparative genomics approach based upon bioinformatics and PCR-based cloning of cDNA and genomic DNA to characterise the marsupial *ELP* and eutherian *CTI* genes and investigate their evolutionary history.

## Results

### *ELP*/*CTI* evolved from a common ancestral gene

To determine whether the marsupial *ELP* gene was present in other species, we used multiple approaches. We cloned the *ELP* genes of the koala and fat-tailed dunnart and isolated tammar *ELP* from a genomic library. *ELP*/*CTI* transcripts were cloned from the mammary gland of the cow, opossum and fat-tailed dunnart and the dog *CTI* transcript was cloned from epithelial cells isolated from canine colostrum. We performed BLAST searches of genomic databases (Ensembl, Release 62, April 2011 [49], NCBI GenBank nr and WGS [54] and UCSC [55]), using a cut-off of E-value ≤ 1e-8 (nucleotides) and E-value ≤ 1e-17 (proteins). To further refine the identification of *ELP*/*CTI* orthologues based upon protein sequence, we also compared gene structures (where possible) to identify genes with a similar three-exon structure to *ELP*/*CTI*. Based upon these methods, no genes orthologous to marsupial *ELP*/eutherian *CTI* were present in fish (Zebrafish, Tiger and green spotted puffers, Three-spined stickleback), birds (chicken, zebra finch), amphibian (African clawed frog), reptile (Green anole lizard), monotreme (platypus), nor sea squirts, fruit fly, nematode (*Caenorhabditis elegans*) or yeast. However, many of the current genomes available provide only low sequence coverage (e.g. anole lizard, 2x; green spotted pufferfish, 2.5x; chicken, zebra finch and platypus, 6x; elephant, 7x). Many assemblies are also incomplete (contain gaps) and may contain incorrect assemblies. Hence it is possible that *ELP*/*CTI* orthologues may be identified within these genomes with future improvements in sequence coverage and assemblies.

The *CTI* gene was present in the Laurasiatherian orders Cetartiodactyla (cow, pig, common bottle-nosed dolphin) and Carnivora (dog, cat, Giant panda). However, based upon current genome assemblies, it is a pseudogene in Afrotheria, Xenarthra, Euarchontoglires and the Laurasiatherian orders Chiroptera and Perissodactyla.

**Figure 1:**
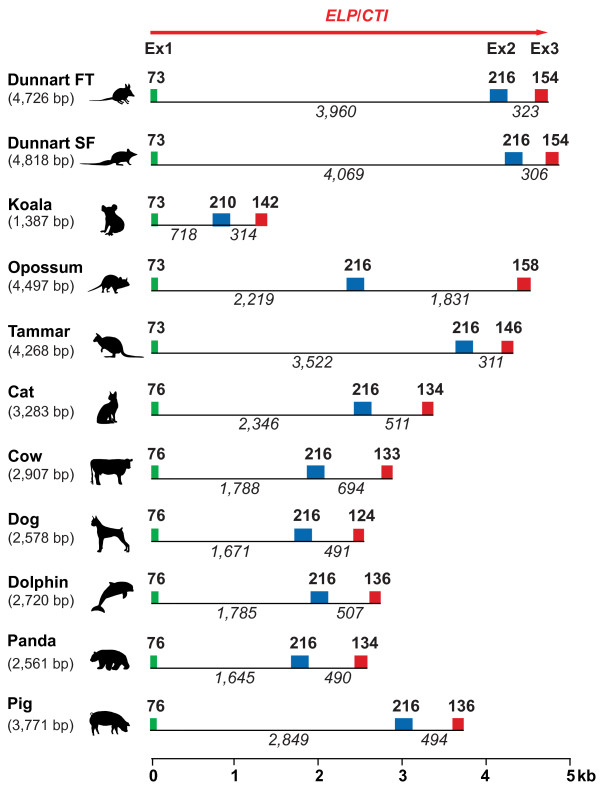
**Structure of the marsupial*ELP*and eutherian*CTI*genes.** The *ELP/CTI* genes of the stripe-faced (SF) dunnart (*Sminthopsis macroura*) [GenBank: AC186006], fat-tailed (FT) dunnart (*Sminthopsis crassicaudata*) [GenBank: JN191336], koala (*Phascolarctos cinereus*) [GenBank: JN191337], opossum (*Monodelphis domestica*) [GenBank: BK008085], tammar (*Macropus eugenii*) [GenBank: JN191335], cat (*Felis catus*, Abyssinian domestic cat) [GenBank: BK008083], cow (*Bos Taurus*, Hereford Breed) [Ensembl: ENSBTAG00000016127], dog (*Canis familiaris*, Boxer breed) [GenBank: BK008082], dolphin (*Tursiops truncatus*) [GenBank: BK008086], pig (*Sus scrofa domestica*) [Ensembl: F1SD34_PIG (ENSSSCG00000007398)] and Giant panda (*Ailuropoda melanoleuca*) [GenBank: BK008084] have 3 exons and 2 introns. Gene size is indicated within brackets and refers to the number of nucleotides from the putative translation start (ATG, exon 1) to the polyadenylation signal (AATAAA, inclusive, exon 3). Exons are colour-coded: exon 1 (green rectangle), the Kunitz domain-encoding exon 2 (blue) and exon 3 (red) and exon size is indicated in bold text. Intron sizes are italicised. The horizontal scale bar indicates the relative sizes of the *ELP*/*CTI* genes (kb), with the putative translation start site (ATG) of all sequences aligned with the origin (0 kb). Genes are drawn approximately to scale.

The mammalian *ELP*/*CTI* gene was composed of 3 exons and 2 introns (Figure 1). The marsupial *ELP* gene ranged from ~1.4 kb for the koala to ~4.8 kb for the stripe faced dunnart, whilst eutherian *ELP* spanned from ~2.5 kb for the panda to ~3.8 kb for the pig. *ELP* exon 1 and 2 sizes respectively were highly conserved across all mammals (Figure 1). Exon 1 encoded the putative signal peptide and the first four amino acids at the N-terminus of the protein. The 216 bp exon 2 (with the exception of the koala, 210 bp) encoded the remainder of the N-terminal region, plus a single BPTI-Kunitz domain towards its 3'-end. *ELP*/*CTI* exon 3 differed most and encoded a maximum of seven amino acids. The *ELP/CTI* transcripts (putative translation start site to the polyadenylation signal, inclusive) were short. Marsupial *ELP* and eutherian *CTI* transcripts ranged from 425–447 bp and 416–428 bp respectively and shared 56.1%-63.6% similarity at the nucleotide level (Additional file 2: Figure S1; Additional file 3: Tables S2A, S2B). A highly conserved marsupial-specific region (87%-100%) was also identified within the *ELP* 3'-UTR (nt 420–475, Additional file 2: Figure S1; Additional file 3: Table S2C).

**Figure 2:**
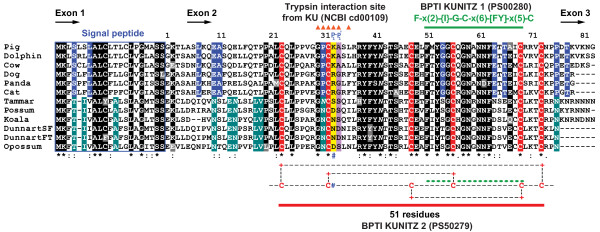
**Alignment of the marsupial ELP and eutherian CTI precursor proteins.** The nucleotide sequences of the *ELP*/*CTI* mRNA transcripts of the following species were conceptually translated and aligned with ClustalW2: tammar [GenBank: JN191338; UniProtKB/Swiss-Prot: O62845 (mature protein)], brushtail possum [GenBank: U34208], fat-tailed dunnart (FT) [GenBank: JN191339], opossum [GenBank: JN191340], cow (Holstein-Friesian breed) [GenBank: JN191341] and dog [GenBank: JN191342]. The stripe-faced dunnart (SF) [GenBank: AC186006], koala [GenBank: JN191337], cat [GenBank: BK008083], pig [Ensembl: F1SD34_PIG (ENSSSCT00000008098)], dolphin [GenBank: BK008086], and panda [GenBank: BK008084] *ELP*/*CTI* genes were conceptually spliced based upon conserved splice sites and translated. Amino acid residues are numbered based upon the start (N-terminus) of the mature ELP/CTI peptides. Black shading indicates nucleotide residues common to at least 10 of the species and grey, the remainder that differ. The six conserved cysteine residues (C1-C6, C2-C4 and C3-C5), which form the three disulphide bonds and produce a globular protein are shaded red. Teal shading indicates amino acids common to marsupials and blue, those common to eutherians. The location of exons is indicated by arrows. The predicted signal peptides are boxed (blue). The BPTI KUNITZ 1 and 2 motifs are indicated (green and red bars respectively) and the putative trypsin interaction site from the KU motif (NCBI cd00109) is depicted by orange triangles. The putative P_1_ and P_1_' reactive site residues are shaded yellow and purple respectively. Italicised asparagine (*N*) residues indicate predicted sites of post-translational N-glycosylation. Conservation between groups of amino acids with strongly similar properties, i.e., scoring > 0.5 in the Gonnet PAM 250 matrix is indicated (:). Conservation between groups of amino acids with weakly similar properties (scoring < 0.5 in the Gonnet PAM 250 matrix) is also noted (.). Gaps within the alignment are indicated (−).

Based upon signal peptide analysis [56], the putative ELP/CTI peptides identified in this study were predicted to be secreted in milk, as for tammar and possum ELP and bovine CTI [20,25,26,31]. The mature ELP and CTI peptides shared 44.6%-62.2% similarity (Table 1; Additional file 4: Table S3A). In addition, the conservation of the two Kunitz domain motifs in all species suggested they may inhibit the S1 family of serine endopeptidases like many other members of the BPTI-Kunitz family [43,44]. The BPTI KUNITZ 2 motif [C1-C6, C2-C4 and C3-C5, Prosite: PS00280] indicates the 3 disulphide bonds which determine the structure of the domain (Figure 2). This motif spanned the entire 51 amino acid Kunitz domain (aa 23–73, C23-C73, C32-C56 and C48-C69, Figure 2). The second shorter motif BPTI KUNITZ 1 [F-x(2)-{I}-G-C-x(6)-[FY]-x(5)-C; where x represents any residue, those within square brackets are permitted, but those within curly brackets are not, Prosite: PS00280] was located within BPTI KUNITZ 2 (aa 51–69, Figure 2). A putative trypsin interaction site within the Kunitz domain (from KU NCBI cd00109) [57], is also depicted (aa 30–34, 36, Figure 2).

**Table 1:** **Homology between and within the marsupial ELP and eutherian CTI peptides**^**1**^

**Species comparisons**	**Signal peptide**	**Mature peptide**	**N-terminus**	**Kunitz motif2 (51 aa)**	**Kunitz motif1 (19 aa)**	**C-terminus**
**Marsupial ELP**	85 - 95%	67.5 - 100%	59.1 - 100%	76.5 - 100%	84.2 - 100%	20 - 100%
**Eutherian CTI**	57.1 - 90.5%	70.7 - 88.6%	59.1 - 90.9%	76.5 - 94.1%	84.2- 100%	40 - 83.3%
**Marsupial ELP vs Eutherian CTI**	57.1 - 81.0%	44.6 - 62.2%	18.2 -59.1%	54.9 - 68.6%	63.2 - 73.7%	10 - 60%

Pairwise amino acids similarities were calculated using MatGAT 2.01 (BLOSUM62 matrix).

^1^Refer to Additional file 4: Tables S3 for individual species comparisons.

Conserved amino acid residues within a protein provide an indication of sites essential for its structure and biological function. Comparison of the marsupial ELP and eutherian CTI precursor proteins showed that the signal peptide (57.1%-81.0% similarity), the 51 aa BPTI KUNITZ 2 motif (54.9%-68.6%), plus the shorter 19 aa BPTI KUNITZ 1 motif within it (63.2%-73.7%) were conserved. However, the 20–22 residue linear chain of the mature ELP/CTI N-terminus had marsupial-specific and eutherian-specific homology (59.1%-100%, Table 1; Additional file 4: Tables S3B, S3C, S3D, S3E). Conservation of the short (3–10 residue) C-terminus was variable (Additional file 4: Table S3F). This was in part due to the use of different stop codons in *ELP*/*CTI* transcripts across divergent species. The opossum and dunnart ELP proteins were truncated at the end of exon 2, with the stop codon encoded by one nucleotide in exon 2 and two in exon 3 (nt 323–325 inclusive; Additional file 2: Figure S1). For all other species, two different stop codons within exon 3 were used. For the panda, cat and dog, the TAA stop codon (nt 333–335) was used. However, for the pig, cow, dolphin and the remainder of the marsupials, the equivalent TGA stop codon (nt 344–346 inclusive) was used.

Surprisingly, there was little conservation of the amino acid residue type (physiochemical properties) at the P_1_ reactive site within the Kunitz domain (residue 33, Figure 2). Although the P_1_ residue type (basic amino acid with a positively charged side chain) was conserved amongst eutherians: K (lysine) for the pig, cow and dolphin and R (arginine) for the cat, dog and panda, this was not so for marsupials. The opossum and possum ELP P_1_ residue was acidic with a negatively charged side chain (D, aspartate). However, the P_1_ residue for tammar (S, serine) and the koala and dunnarts (N, asparagine) was polar with uncharged side chains.

Although P_1_ residues differed, all ELP/CTI peptides were predicted to be N-glycosylated at asparagine-42, consistent for bovine CTI [58] and therefore should be larger than their predicted masses (8.6 to 9.6 kDa, data not shown).

### Selective pressure acting upon marsupial ELP and eutherian CTI

The evolutionary selection pressure acting upon different regions of the protein-coding marsupial *ELP* and eutherian *CTI* transcripts was determined by dN/dS analysis (Table 2). The dN/dS ratio measures the number of non-synonymous changes per non-synonymous site (those which produce amino acid substitutions) compared to the number of synonymous changes per synonymous site (no amino acid change) [59,60]. A ratio of dN/dS = 1 suggests a neutral condition, with nucleotide changes accumulating in the absence of selection pressure, i.e. both dN and dS occur at the same rates. dN/dS < 1 indicates purifying selection, with amino acid changes not tolerated. In contrast, dN/dS > 1 is indicative of positive Darwinian selection for amino acid changes [59,61].

**Table 2:** Average rates of synonymous (dS) and non-synonymous (dN) substitutions occurring in marsupial ELP and eutherian CTI

**ELP/CTI protein-coding** **region**		**dN**	**SE**	**dS**	**SE**	**dN/dS** **Ratio**	**(a) Neutral selection test (dN ≠ dS)**^+^*	**(b) Purifying** **selection test (dN < dS)**^+^*	**(c) Positive selection test (dN > dS)**^+^*
**Precursor protein**	***Marsupials***	0.145	0.022	0.190	0.033	0.763	0.256 (NS^‡^)	0.117 (NS)	1.000 (NS)
	***Eutherians***	0.194	0.026	0.225	0.033	0.862	0.232 (NS)	0.472 (NS)	1.000 (NS)
**Mature protein**	***Marsupials***	0.166	0.026	0.185	0.036	0.897	0.653 (NS)	0.334 (NS)	1.000 (NS)
	***Eutherians***	0.186	0.028	0.242	0.039	0.786	0.273 (NS)	0.130 (NS)	1.000 (NS)
**Signal peptide**	***Marsupials***	0.071	0.029	0.226	0.094	0.314	0.133 (NS)	0.064 (NS)	1.000 (NS)
	***Eutherians***	0.225	0.072	0.165	0.069	1.36	0.451 (NS)	1.000 (NS)	0.224 (NS)
**N-terminus**	***Marsupials***	0.240	0.064	0.116	0.048	2.07	0.064 (NS)	1.000 (NS)	0.041*
	***Eutherians***	0.242	0.050	0.224	0.065	1.08	0.842 (NS)	1.000 (NS)	0.424 (NS)
**BTPI KUNITZ 2**^**#**^	***Marsupials***	0.146	0.031	0.224	0.052	0.651	0.215 (NS)	0.101 (NS)	1.000 (NS)
	***Eutherians***	0.162	0.035	0.243	0.054	0.667	0.200 (NS)	0.105 (NS)	1.000 (NS)
**BPTI KUNITZ 1**^**~**^	***Marsupials***	0.095	0.030	0.223	0.098	0.426	0.212 (NS)	0.103 (NS)	1.000 (NS)
	***Eutherians***	0.066	0.026	0.264	0.110	0.250	0.122 (NS)	0.046*	1.000 (NS)
**Trypsin interaction site**^**^**^	***Marsupials***	0.230	0.136	0.323	0.181	0.712	0.740 (NS)	0.363 (NS)	1.000 (NS)
	***Eutherians***	0.175	0.093	0.228	0.131	0.768	0.689 (NS)	0.345 (NS)	1.000 (NS)

^#^PS50279 153 nt, 51 aa.

^~^PS00280 57 nt, 19 aa.

^^^18 nt, 6 aa site from KU (NCBI cd00109).

^+^Codon based Z-tests in MEGA5.

*p < 0.05.

^‡^*NS* not significant.

The protein-coding marsupial *ELP* and eutherian *CTI* transcripts and regions within them generally exhibited a trend towards purifying selection, with a dN/dS ratio <1 (Table 2). However, based upon codon-based Z-tests, only the eutherian *CTI* BPTI KUNITZ 1 motif (57 nt encoding 19 amino acids) was found to be undergoing purifying selection (p < 0.05). Although the regions encoding the marsupial BPTI KUNITZ 1 motif (p = 0.103) and the marsupial and eutherian BPTI KUNITZ 2 motifs (p = 0.101 and p = 0.105 respectively) exhibited a strong trend towards purifying selection, the test values (dN < dS) were not significant. This tendency was also consistent for the putative trypsin interaction site. In contrast, three regions of the *ELP*/*CTI* transcripts showed a trend towards positive selection (dN/dS > 1). These included the regions encoding the ELP/CTI N-terminus and the eutherian CTI signal peptide. However, based upon codon-based Z-tests (dN > dS), only the eutherian CTI signal peptide (p < 0.05) was undergoing positive selection.

### Marsupial *ELP* and eutherian *CTI* share common flanking genes

**Figure 3:**
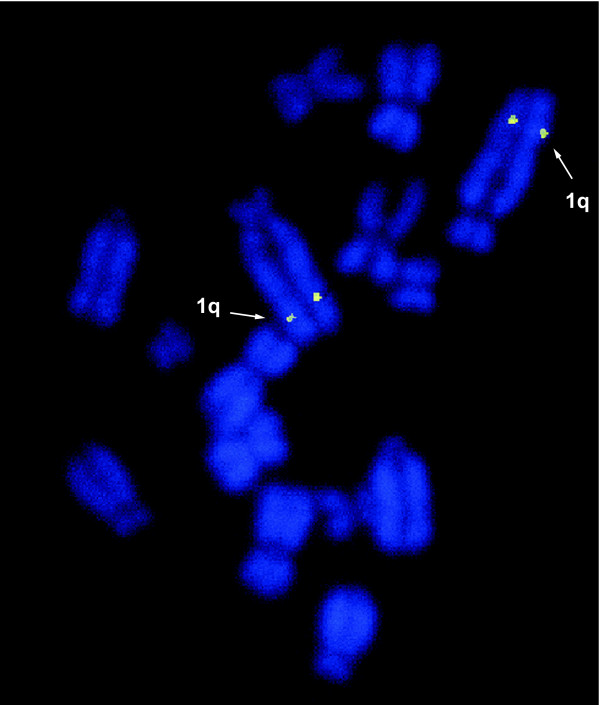
**Localisation of the tammar*ELP*gene to*Macropus eugenii*chromosome 1q using FISH.**

**Figure 4:**
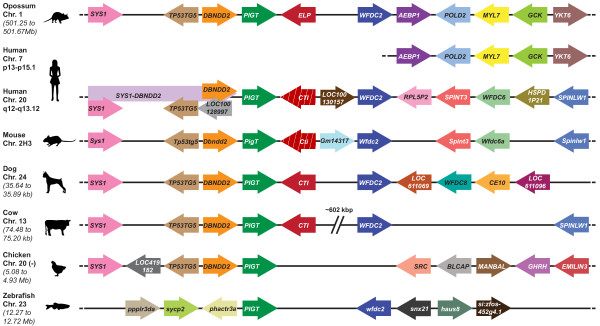
**Chromosomal location of the*ELP/CTI*gene in different species.** The *ELP/CTI* gene was located within a syntenic block on opossum Chr. 1 (~501.34 Mb), human Chr. 20q12-13.12, mouse Chr. 2 H3, dog chr. 24 (~35.7 Mb) and cow Chr. 13 (~74.5 Mb) [49,55]. However, *ELP/CTI* was reduced to a pseudogene in the human and mouse (red arrow, white diagonal stripes) and was absent in the chicken and zebrafish. The *ELP/CTI* gene was located on the reverse strand and was generally flanked by one, or both of the single-copy genes *PIGT* and *WFDC2.* The region upstream of *PIGT* was conserved in mammals and the chicken and included the *SYS1* [Golgi-localized integral membrane protein homolog (*S. cerevisiae*)], *TP53TG5* (*TP53-target gene 5 protein*), and *DBNDD2dysbindin* (*dystrobrevin binding protein 1*) *domain containing 2* genes. However, a chromosomal breakpoint was located downstream from the eutherian *WFDC2* gene. Opossum chromosome 1 contained the *AEBP1* (*Adipocyte enhancer binding protein 1*), *POLD2polymerase (DNA directed), delta 2, regulatory subunit 50 kDa*, *MYL7* (*myosin, light chain 7*, regulatory) and *YKT6YKT6* v-SNARE homolog (*S. cerevisiae*)] genes and was orthologous to human chromosome 7p13-p15.1. In contrast, the eutherian chromosomes contained a number of genes which encoded Kunitz and/or WAP domains. These included *SPINT3SPINLW1WFDC8* and *WFDC6*, which were likely to have arisen by gene and domain duplications [62]. Notably, there was an insert of ~602 kb between bovine *CTI* and *WFDC2*. Arrows indicate the arrangement and orientation of genes and are not drawn to scale.

In order to confirm that the marsupial *ELP* and eutherian *CTI* genes were orthologous, we characterised the location and arrangement of *ELP/CTI* and its flanking genes. We used fluorescence *in situ* hybridisation to map tammar *ELP* to chromosome 1q (Figure 3). The *ELP/CTI* gene was located on a syntenic segment in the marsupial (stripe-faced dunnart [27] and opossum) and eutherian genomes [49,55] and was generally flanked by one or both of the single-copy genes *phosphatidyl inositol glycan, class T* (*PIGT*) and *WAP four disulphide core domain 2* (*WFDC2*), confirming they were true orthologues (Figure 4).

The *PIGTWFDC2* region of bovine chromosome 13 (~74.51-75.14 Mb) was unique. Bovine *CTI* was adjacent to *PIGT*, but there was an insertion of ~602 kb between the *CTI* and *WFDC2* genes [49,55] (data not shown). This region included 7 Artiodactyla-specific Kunitz domain-encoding genes including *PTISTI*, plus the five placenta-specific *TKDP1-TKDP5* genes inclusive [50,63]. Furthermore, the *SPINLW1* gene which contains both a Kunitz and a WAP domain and the eutherian-specific *SPINT4* gene were located a further ~38 kb and ~90 kb respectively downstream from *WFDC2*[49,55] (data not shown). As mentioned previously, these genes, with the exception of *SPINLW1* and the *TKDP*s, share a similar 3-exon structure. However, the *TKDP*s differ due to the likely “exonisation” of an intron and its subsequent duplication to produce a variable number of tripartite N-domains between the exon encoding the signal peptide and the Kunitz domain [50,51].

### *CTI* has been lost in some eutherians

**Figure 5:**
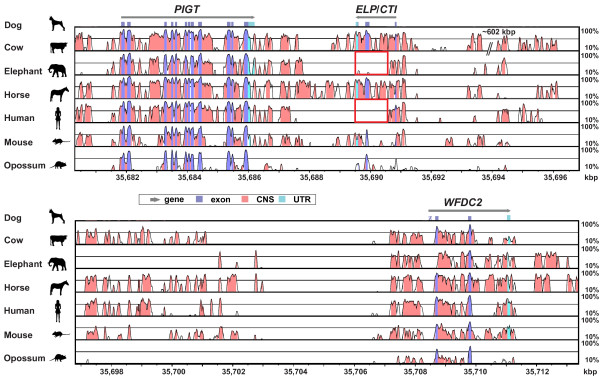
**VISTA plot of pairwise alignments for selected mammals in the region containing the*PIGT*,*ELP/CTI*and*WFDC2*genes.** Sequence homology within the *PIGT-ELP/CTI-WFDC2* region of the dog, cow, elephant, horse, human, mouse and opossum genomes was determined with mVISTA [64]. The dog sequence was used as the reference sequence (horizontal axis, dog chromosome 24 numbering). Grey horizontal arrows indicate gene location and direction of transcription. Blue rectangles indicate coding exons and untranslated regions (UTRs) of the gene are depicted by light green rectangles. Exon 1 of canine *WFDC2* was missing (gap in the current assembly) from the dog genome and is indicated by a blue rectangle with diagonal white stripes. The right axis indicates the percentage identity within a 100 bp window for each pairwise comparison, ranging from 10% to 100%. Regions sharing greater than 25% identity are shaded and the black horizontal line indicates 70% identity. The region containing the Kunitz domain-encoding *ELP/CTI* exon 2 was conserved in the cow, horse, mouse and opossum, but was absent in the elephant and human *CTI* genes (red boxes).

Using the canine sequence as the basis for mVISTA comparative analysis [64], the region between the *PIGT* and *WFDC2* genes was examined using the available genome assemblies - which have variable sequence coverage, contain gaps and may contain misassembled sequences. Whilst the *ELP*/*CTI* gene was present in some mammals, it appeared to have become a disrupted pseudogene in others such as the African Savanna elephant and human (Figure 5). Exon 1 of the elephant and human *CTI* genes (signal- and pro-peptide) was present, but exon 2 (Kunitz domain) and exon 3 (C-terminus) were absent (red boxes, Figure 5), suggesting they had been excised or transposed, whilst the horse and mouse *CTI* genes initially appeared intact.

A closer examination of the nucleotide sequence between *PIGT* and *WFDC2* in these and other species using the Ensembl and UCSC genome databases revealed that different mutations had most likely disrupted the *CTI* gene. Exon 1 was disrupted in the elephant, Hoffmann's two-toed sloth (*Choloepus hoffmanni*), armadillo (*Dasypus novemcinctus*), human and other primates and horse, with exon 2 (Kunitz domain) also excised for these species, apart from the horse. Additional file 5: Figure S2A (i) depicts a nucleotide alignment of the functional/protein-coding dog *CTI* exon 1 compared with the putative disrupted *CTI* exon 1 of the elephant, sloth, human and horse. Additional file 5: Figure S2A (ii) shows the translated sequences to highlight mutations and/or deletions within the signal peptide region of CTI. The deletion of two nucleotides within human *CTI* exon 1 would produce a frame-shift (as depicted by the +1 and +2 reading frames). *CTI* exon 2 of the mouse, rat, large flying fox (*Pteropus vampyrus*) and horse also appeared to have been disrupted by deletions resulting in frame-shifts when compared to the functional/protein-coding dog *CTI* exon 2. The disruption of the protein-coding region of equine *CTI* exons 1 and 2 by at least one mutation and one deletion respectively would produce a frame-shift, suggested these were a recent occurrence (Additional file 5: Figure S2B (ii)).

### Transposable elements within the *ELP/CTI* genes

Transposable elements integrate randomly into the genome, so the probability of the same element(s) integrating independently into orthologous positions in different species is extremely low. They therefore act as genetic markers and can be used to determine the phylogenetic relationship between genes and species [65]. Further evidence that marsupial *ELP* and eutherian *CTI* evolved from a common ancestral gene was provided by CENSOR retrotransposon analysis [66] (Additional file 6: Figure S3). Retroelements of conserved fragment size and orientation were located within the *PIGTELP/CTI* region. However, the elephant and human which appear to have lost *CTI* exons 2 and 3, had also lost retrotransposons in the corresponding region, but gained a MER5A element.

### Bovine *CTI*, *PTI*, *STI* and the *TKDPs* share a common ancestral gene

**Figure 6:**
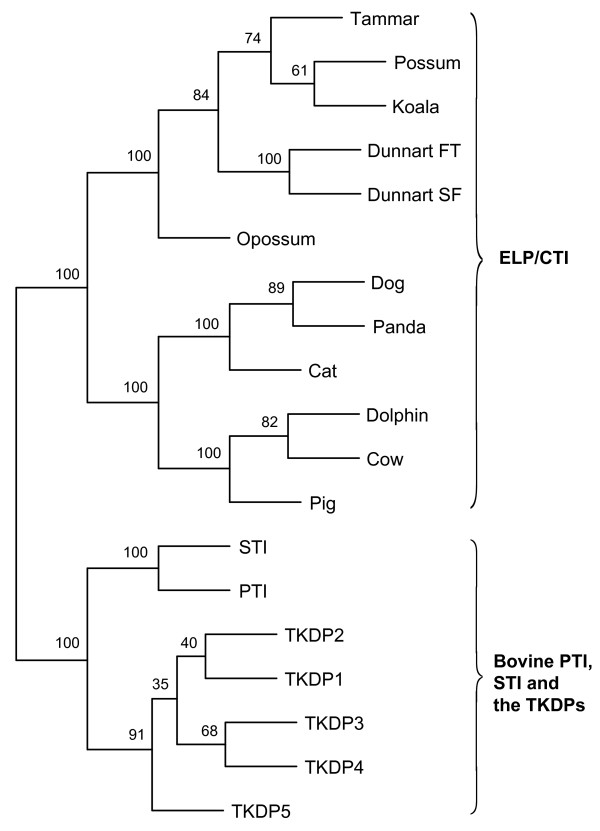
**A phylogenetic tree of*ELP*/*CTI*and the*CTI*-like bovine*PTI*,*STI*and*TKDP1*,*2*,*3*,*4*and*5*family.** The evolutionary relationship between the protein-coding regions of the marsupial *ELP*, eutherian *CTI* and bovine *TKDP1-5*, *PTI* and *STI* transcripts was determined by maximum likelihood analysis using a molecular clock assumption. The bovine *SLPI* transcript was used as an outgroup (data not shown). Two main groups were formed: 1. mammalian *ELP*/*CTI* and 2. bovine *CTI*, *PTI* and the *TKDP*s. Numbers at branch points indicate confidence levels as determined by bootstrap values (100 replicates). Phylogenetic trees were produced with Phylip software version 3.69. Transcripts were aligned with MUSCLE and boostrapped values generated with SEQBOOT. Maximum likelihood trees were generated with DNAMLK using a transition/transversion ratio of 1.34, a Gamma distribution shape of 1.39 with 5 Hidden Markov Model categories, global rearrangements and with a randomised input order jumbled once. The protein-coding regions of the following transcripts were used in the analysis: *ELP*/*CTI*, tammar [GenBank: JN191338], fat-tailed dunnart [GenBank: JN191339], stripe-faced dunnart [GenBank: AC186006], koala [GenBank: JN191337] opossum [GenBank: JN191340], brushtail possum, cow [GenBank: JN191341], dog [GenBank: JN191342], cat [GenBank: BK008083], pig [Ensembl: F1SD34_PIG (ENSSSCT00000008098)], Giant panda [GenBank: BK008084], and Common bottlenose dolphin [GenBank: BK008086], and the following bovine transcripts: *PTI* [GenBank: NM_001001554], *STI* [GenBank: NM_205786], *TKDP1* [GenBank: NM_205776], *TKDP2* [GenBank: NM_001012683], *TKDP3* [GenBank: XM_584746], *TKDP4* [GenBank: NM_205775], and *TKDP5* [GenBank: XM_614808] and *SLPI* [GenBank: NM_001098865].

The location of the 8 Kunitz-domain encoding genes (including *CTI*) on bovine chromosome 13 between the *PIGT* and *WFDC2* genes and the Artiodactyla-specific distribution of *PTISTI* and *TKDP1-5* (cow and sheep [51,63]) suggested they may have evolved from *CTI*. This hypothesis was supported by phylogenetic analysis of the protein-coding regions of the mammalian *ELP*/*CTI*, bovine *PTISTI* and *TKDP1-5* transcripts, with bovine *SLPI* used as an outgroup root (*SLPI* omitted, Figure 6). Several different methods in PHYLIP were used to determine the evolutionary relationships. These included the character-based maximum-likelihood (with/without a molecular clock) and maximum parsimony, as well as distance-based analysis (Fitch-Margoliash tree method using the Kimura distance model of nucleotide substitution). Trees were evaluated using the bootstrap method (100 replicates). Of the algorithms used, the maximum likelihood method using a molecular clock assumption, which assumes a constant evolutionary rate for all species, produced a tree with the highest bootstrap values. Huttley and colleagues [67] have shown that the eutherian nucleotide substitution rates are ~30% slower than for marsupials. However, all methods produced consensus trees which consistently separated the 19 sequences into the two groups depicted (Figure 6). The hypothesis that bovine *CTI* was the ancestral gene for \bovine *PTISTI* and *TKDP1-5* was supported by both an alignment of precursor proteins and phylogenetic analysis of *CTIPTISTITKDP1-5* and the *SPINT4* protein-coding transcripts (Additional file 7: Figure S4; Additional file 8: Figure S5). Interestingly, the size of the Kunitz domain-encoding exon varied. Whilst the bovine *CTI* exon was 216 bp, those of the TKDPs were 196 bp, with 192 bp for *PTI* and *STI* and 175 bp for *SPINT4*. Furthermore, apart from CTI and SPINT4, none of the Kunitz domains were predicted to be N-glycosylated. Additional evidence of the evolutionary history of the *CTIPTISTI* and *TKDP1-5* genes was provided by mVISTA (Additional file 9: Figures S6A and S5B (i-viii) and CENSOR analysis (Additional file 10: Figure S7; Additional file 11: Table S4).

### Tammar *ELP* expression is up-regulated at parturition and is mammary-specific

**Figure 7:**
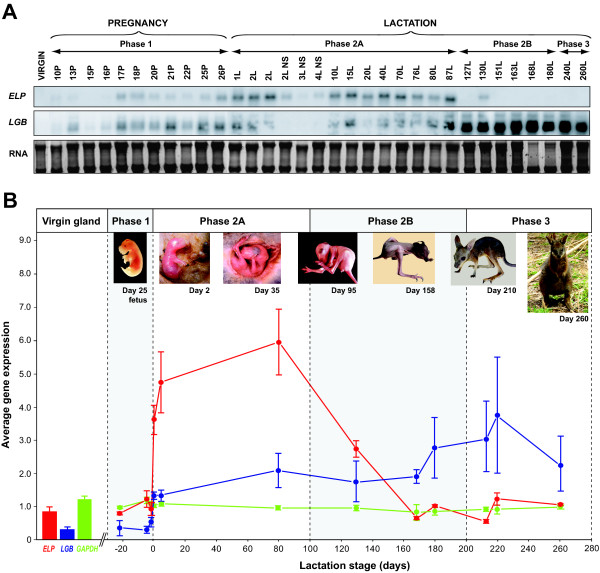
***ELP*expression in the tammar mammary gland throughout the reproductive cycle. A.** Northern analysis of total RNA (10 μg) extracted from the mammary glands of tammar wallabies during pregnancy (P, Phase 1) and lactation (L, Phase 2A, Phase 2B and Phase 3), from non-sucked (NS) glands and from a virgin female (~220 days of age). Tammar *ELP* expression was undetected in the virgin gland, minimal during pregnancy (Phase 1) and then induced at parturition and expressed during early lactation (Phase 2A). *ELP* was down-regulated at mid-lactation (Phase 2B), consistent with [13,20,21]. *ELP* transcripts were not detected in Phase 3. *ELP* expression also declined postpartum in non-sucked glands. Tammar *LGB* was used as a positive control for lactation and exhibited a similar expression pattern to *ELP*, but with *LGB* expression increased (but not significantly so) during Phases 2B and 3, as reported previously [13,68,69]. Ribosomal RNA bands indicate RNA integrity and loading. **B.** Microarray analysis of the tammar mammary gland [ArrayExpress: E-MTAB-1057] supported the quantitative analysis of Northern blot (data not shown) and microarray data reported by [69]. Expression of the *ELP* and *LGB* milk protein genes and the housekeeping gene *GAPDH* (glyceraldehyde 3-phosphate dehydrogenase) is depicted as average normalised raw intensity based upon the expression n = 3, 7 and 2 clones on each microarray respectively ± SEM (Additional file 12: Table S5). Whilst *ELP* (red) and *LGB* (blue) expression differed during the reproductive cycle, *GAPDH* (green) expression was constant.

Northern analysis showed that tammar *ELP* was up-regulated at parturition, consistent with brushtail possum *ELP*[28] (Figure 7A). *ELP* transcripts were detected in the tammar mammary gland from ~ day 17 of pregnancy onwards, throughout early lactation (Phase 2A) until ~ day 87 of lactation. *ELP* was then down-regulated to minimal levels for the remainder of lactation. This was consistent with a previous study of late Phase 2A/Phase 2B mammary tissues, but the precise timing of *ELP* gene induction was not investigated [13,20,21]. Neither *ELP*, nor *LGB* was expressed in the virgin mammary gland and both genes were down-regulated postpartum in the non-sucked glands (Figure 7A), as in the brushtail possum [28].

*LGB* expression peaked in the mammary gland during Phase 3, consistent with [68].

**Figure 8:**
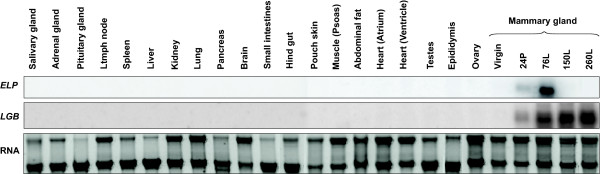
**Tammar*ELP*expression was specific to the mammary gland.** Northern analysis of total RNA (10 μg) extracted from assorted tammar tissues indicated that both *ELP* and *LGB* expression were specific to the mammary gland. Ribosomal RNA bands indicate RNA integrity and loading.

Although cDNA microarray analysis of the tammar mammary gland (Figure 7B; Additional file 12: Table S5) was based upon comparative expression levels rather than actual transcript levels, the data was consistent with quantitative analysis of the Northern blot (data not shown) and microarray data reported by [69]. Lastly, Northern analysis of assorted tammar tissue samples indicated that expression of *ELP* and *LGB* was mammary gland-specific (Figure 8), unlike the ubiquitously expressed *cystatin C* (*CST3*) gene (data not shown).

## Discussion

**Figure 9:**
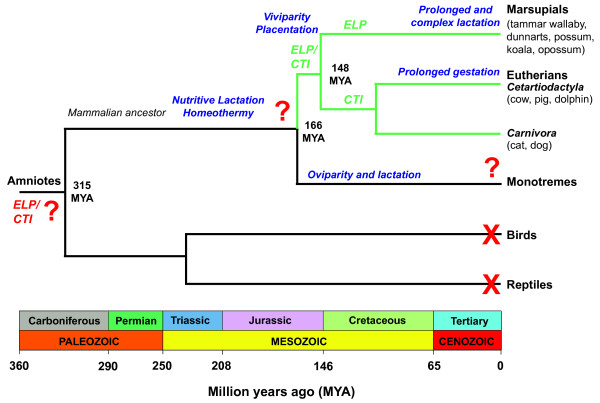
**Evolution of the*ELP*/*CTI*gene in therians.** Tree depicting the relationship between the amniotes: birds, reptiles, monotremes, marsupials and eutherians [1,3,70,71] and the distribution of the *ELP*/*CTI* gene. The divergence times used are based upon the analysis by Bininda-Emonds and colleagues [1]. Extant species which have a functional *ELP*/*CTI* gene are indicated by green tree branches. Extant species in which the *ELP*/*CTI* gene has not been detected are indicated by a red cross. Lineages on the tree for which the presence or absence of the *ELP*/*CTI* gene remains inconclusive are indicated by a red question mark. Based upon current analyses, the functional *ELP*/*CTI* gene evolved at least 130 million years ago (MYA) and has been retained by extant marsupials and the Laurasiatherian orders Cetartiodactyla and Carnivora. Whether the *ELP*/*CTI* gene is present in monotremes is unknown.

*ELP* was originally thought to be a marsupial-specific gene [19]. However, we have shown that the marsupial *ELP* and eutherian *CTI* genes evolved from a common therian ancestral gene (Figure 9). Mammalian *ELP/CTI* was generally flanked by one or both of the single copy *PIGT* and *WFDC2* genes in a region that was syntenic to that of other mammals. The conserved genomic structure of 3 exons and 2 introns and homologous transposable element fragments confirmed that *ELP* and *CTI* were true orthologues. *CTI* was also identified as the putative ancestral gene of the ruminant-specific *PTISTI* and *TKDP1-5* genes. Based upon current genome sequencing and assemblies, *ELP/CTI* was not found in birds, fish, reptiles, nor amphibians, suggesting the gene was present in the therian ancestor before the divergence of marsupials and eutherians at least 130 million years ago [1,2,70].

### Mammalian *ELP/CTI* and the evolution of bovine *PTI*, *STI* and the *TKDP*s

The Kunitz-type inhibitor domain has been duplicated many times throughout evolutionary history [38]. This was no more evident than for the region of bovine chromosome 13 on which *CTI* and the 7 *CTI*-like genes were located. The *PTISTI* and *TKDP1-5* genes were specific to the order Cetartiodactyla, sub-order Ruminantia [50,51,63,72], strong evidence they evolved from *CTI* after the divergence of the Ruminantia ~25-35 MYA [1]. The *CTIPTI* and *STI* genes had a similar 3-exon structure and conserved regions within both coding and non-coding segments. The *PTI* and *STI* genes and proteins were homologous and almost certainly arose by gene duplication [73]. However, the *TKDP1-5* genes had one or more additional exons inserted between the signal- and pro-peptide-encoding and Kunitz domain-encoding exons (equivalent to intron 1 of *CTIPTI* and *STI*) resulting in an expansion to 4 (*TKDP5*), 6 (*TKDP23* and *4*) and 12 exons (*TKDP1*) [50,51,72]. These added exons encode tripartite N-domains which had no similarity to database sequences or motifs and evolved recently due to the “exonization” of an intron within an active MER retrotransposon and its subsequent duplication [50,63]. These elements have been associated with genetic rearrangements and deletions [74]. This may explain the excision of *CTI* exons 2 (Kunitz domain) and 3 (C terminus) for the elephant and primates, based upon current genome sequencing and assemblies.

### Lack of conservation of the ELP/CTI putative P_1_ reactive site residue

All putative ELP/CTI peptides were predicted to be secreted and shared a conserved single 51 amino acid Kunitz domain. The conserved location of the 6 cysteine residues which form three disulphide bonds suggested ELP/CTI would, like bovine CTI [75] and PTI [46] form a globular protein. However, neither the identity, physiochemical properties of the ELP/CTI P_1_ reactive site residue, the trypsin interaction site, nor the N- and C-terminus of the proteins were conserved. The P_1_ “warhead” residue plays an essential role in the interaction of a Kunitz inhibitor domain with a serine protease and a P_1_ mutation may alter the protease specificity of the Kunitz domain to a particular substrate and the reaction kinetics [48,76]. Kunitz inhibitors with a basic residue, K (Cetartiodactyla) or R (Carnivora) at P_1_ generally inhibit trypsin or trypsin-like serine endopeptidases such as chymotrypsin, pepsin, plasmin and kallikrein *in vitro* (e.g. bovine CTI and PTI) [31,38,77]. However, Kunitz domains with smaller, uncharged residues at P_1_, such as serine, generally inhibit elastase-like proteases (eg. neutrophil elastase) [43,47,76]. In contrast, Kunitz domains with an acidic, negatively-charged P_1_ residue (e.g. TKDP2) exhibit minimal antiprotease activity *in vitro*[72]. Comparison of BPTI Kunitz domains suggested that the marsupial ELP P_1_ amino acids were quite rare [43,49,55]. Furthermore, the absence of purifying selection within the putative ELP/CTI trypsin interaction site and the lack of conservation of P_1_ residues provides intriguing questions as to the role(s) of the marsupial ELP and eutherian CTI proteins *in vivo*.

Not all Kunitz domains act as protease inhibitors [43]. As mentioned previously, snake and spider venoms contain proteins with Kunitz domains [40]. Some domains inhibit trypsin or chymotrypsin via P_1_, whilst others lack anti-protease activity but have neurotoxic effects by acting as potassium channel blockers [41]. Peigneur and colleagues [78] recently reported a sea anemone Kunitz domain protein, APEKTx1 (*Anthopleura elegantissima* potassium channel toxin 1) which had dual functions. It exhibited both trypsin-inhibitor activity and selectively blocked the Kv1.1 type of voltage-gated potassium channels. Furthermore, not all Kunitz protease inhibitors act via the P_1_ residue. The tick anticoagulant peptide (TAP) inhibits Factor X, Factor Xa and thrombin but the reactive site is located towards the N-terminus of the protein, rather than at the P_1_ residue of the Kunitz domain [79].

### ELP/CTI – a conserved N-glycosylation site predicted within the Kunitz domain

All ELP/CTI proteins shared a putative conserved N-glycosylation site within the Kunitz domain at asparagine-42 (asparagine-40 for koala ELP), consistent with the site identified for bovine CTI *in vitro*[58]. The proportion of sugars attached to glycosylated bovine CTI, possum ELP and tammar ELP varies, 25-40% [58,80], 60% [25] and ~47-55% [20,21,26], respectively. However, as the N-glycosylation site occurs at the base of the pear-shaped protein and at the opposite end to the P_1_ site, it is unlikely to affect protease-binding activity [58]. Unlike bovine CTI, the Kunitz domains of neither bovine PTI, STI, nor for the placenta-specific TKDPs are predicted to be N-glycosylated. In fact, very few Kunitz domains are N-glycosylated, or predicted to be so [43,49,55]. The exceptions are SPINT4, SPINLW1, the first Kunitz domains of bikunin and hepatocyte growth factor activator inhibitor, the second domain of tissue factor pathway inhibitor 1, as well as selected sea anemone peptides. The precise effect of N-glycosylation is uncertain, but it may enhance protein hydrophilicity and solubility, reduce proteolysis, influence cell surface signalling and adhesion and affect protein folding, turnover and quality control [81-83]. Furthermore, oligosaccharides may act as soluble receptor analogues for bacterial and viral pathogens, preventing them from attaching to the wall of the intestines, thereby stopping their passage through the gastrointestinal and urinary tracts of the young [84,85].

The lack of conservation of the ELP/CTI N- and C-terminus was intriguing, particularly the positive Darwinian selection (p < 0.05) acting upon the coil-like marsupial ELP N-terminus. In contrast, the eutherian CTI N-terminus tended towards neutral selection. The N- and C-termini of proteins have been associated with sub-cellular targeting, protein-protein and protein-lipid interactions and macromolecular complex formation [86]. The marsupial- and eutherian-specific homology of the mature ELP/CTI N-terminus suggested these regions may have different activities. However, the lack of conservation of the ELP/CTI C-terminus suggested these areas may have species-specific effects. Interestingly, the conservation of the TGA codon used by the tammar, koala, pig, dolphin and cow for all species but the cat (CGA) suggested it was the ancestral *ELP*/*CTI* stop codon, with more recent mutations producing a shortened ELP/CTI C-terminus in some species. Furthermore, a conserved marsupial-specific region within the 3' UTR may regulate *ELP* gene transcription.

ELP/CTI is expressed and secreted in milk during the early lactation/colostrogenesis period only [this study, [20,21,25-28,31,36,37]]. Furthermore, all mammalian neonates have an innate immune system but an immature adaptive immune system and a gut which is yet to undergo maturation or ‘closure’ and is therefore permeable to macromolecules [16,29,87-89]. For the calf, gut maturation occurs 24–36 hr pp [16], whereas for the tammar, this process does not occur until ~200 days pp [87]. Therefore, maternal milk immunoglobulins such as IgG can be passively transferred via colostrum and Phase 2A/2B milk to the gut of the young calf and tammar, respectively, where they are absorbed by the intestines and enter the circulatory system [16,89]. Hence ELP/CTI may enhance the survival of the young by preventing the proteolytic degradation of maternal immunoglobulins [31], or by protecting the young against pathogens [25]. Although sequence comparisons predict the ELP/CTI peptides are likely to inhibit serine endopeptidases, their true function(s) will only be determined through *in vitro* and/or *in vivo* studies.

The importance of local control mechanisms in the regulation of the tammar mammary glands and *ELP* were highlighted in this study. Whilst *ELP* expression proceeds in the sucked gland, the gene is down-regulated and milk production ceases in the non-sucked glands, as for the possum [28]. However, this partitioning of mammary glands and lactation does not occur in eutherians [6]. Marsupial *ELP/*eutherian *CTI* expression was specific to the mammary gland and lactation (Figure 8), unlike the genes that most likely evolved from bovine *CTI*. PTI and STI are produced in mast cells, which have a protective role and are distributed throughout the body to tissues such as the duodenum, pancreas, lung, pituitary gland, spleen and chondrocytes [90]. In contrast, the five bovine TKDPs are differentially expressed in trophoblast cells of the ruminant placenta only during the peri-implantation period, suggesting they have an important role in the maintenance of the conceptus and pregnancy [51,63,72]. Hence, the bovine *PTISTI* and *TKDP1-5* genes have undergone positive (adaptive) selection, changes in tissue-specific expression and function compared to the putative *CTI* ancestral gene, consistent with gene duplication and neofunctionalisation [91,92].

The location of the *CTI* gene in a rapidly evolving region of the eutherian chromosome [51,62] may explain the conversion of *CTI* into a putative pseudogene in Afrotheria (elephant), Xenarthra (sloth, armadillo), Euarchontoglires (humans, primates, rodents) and in selected Laurasiatherians such as the horse and flying fox.

This region included many additional genes with Kunitz and WAP 4-DSC domains [62], unlike for marsupials. It is possible that the role of CTI is fulfilled by one of these genes and hence the loss of the *CTI* gene is tolerated. Alternatively, CTI function may have become non-essential due to physiological changes in selected species. Notably, milk protein gene loss is not common amongst mammals, as genes involved in milk production are generally under negative selection [93]. However, the conservation of the *ELP*/*CTI* gene in marsupials and Laurasiatherian orders Carnivora (dog, cat, dolphin, panda) and Cetartiodactyla (cow, pig) suggests ELP/CTI has an important role in these species.

## Conclusions

Marsupial *ELP* and eutherian *CTI* evolved from a common ancestral gene and encode a milk protein with a single BPTI-Kunitz serine protease inhibitor domain. Although *CTI* was identified as the putative ancestral gene of *PTI*, *STI* and the placenta-specific trophoblast *TKDP1-5* gene family, the origin of the *ELP*/*CTI* gene is inconclusive. *ELP/CTI* expression in the postpartum mammary gland is brief (~24-48 hrs) in eutherians but prolonged in the tammar and other marsupials (up to 100 days). However, this period correlates with the provision of milk to an immuno-incompetent young, suggesting ELP/CTI may play a vital role in immune protection of the young at this time.

## Methods

### Animals

Tammar wallabies (*Macropus eugenii*) were provided from two different marsupial colonies: VIAS (Victorian Institute of Animal Science), DPI (Department of Primary Industries), Attwood, Victoria and The University of Melbourne, Victoria. Animals were kept in open grassy yards with *ad libitum* access to food, water and shelter, using standard animal husbandry conditions in accordance with the National Health and Medical Research Council guidelines [94]. All experiments were approved by the Animal Experimentation Ethics Committees of the Department of Primary Industries and The University of Melbourne.

### Tissues

Tissues (salivary gland, adrenal gland, pituitary gland, lymph node, spleen, liver, kidney, lung, pancreas, brain, small intestines, hind gut, muscle, heart, ovaries) were collected from adult female tammars (n = 2). Mammary glands were also collected from adult females at different stages of pregnancy and lactation (n = 60). Mammary glands from virgin females were collected from tammar pouch young (~220 days of age, n = 3). Testes and epididymides were collected from adult tammar males (n = 2). Tissue samples derived from ear-tagging of a population of koalas (*Phascolarctos cinereus*) located on French Island, Victoria, were donated by Dr. Kath Handasyde and Dr. Emily Hynes from the Department of Zoology, The University of Melbourne. Total RNA extracted from a grey short-tailed opossum (*Monodelphis domestica*) mammary gland from day 15 of lactation (early-lactation) was provided by Dr Denijal Topcic (The University of Melbourne) from animals provided by Professor Norman Saunders (The University of Melbourne). Dr Peter Frappell (Latrobe University) provided fat-tailed dunnart mammary gland tissue from day 37 of lactation (Phase 2) and liver tissue. Dr Amelia Brennan (The University of Melbourne) provided total RNA isolated from the mammary gland of a late-pregnant (~8 months) Holstein-Friesian cow. A small quantity of dog colostrum (~20 μL) from a late-pregnant (~2 weeks prepartum) Labrador in its first pregnancy was also kindly donated by Cate Pooley (The University of Melbourne). All samples were snap frozen in liquid nitrogen and stored at −80°C until use, with the exception of the koala ear punches, which were stored at 4°C.

### RNA extraction and northern analysis

Total RNA was extracted from tissues using the Qiagen RNeasy Midi Kit (Qiagen) and from cells isolated from colostrum using RNAWIZ (Ambion). RNA extracted from cells shed into milk during the lactation process provides a good representation of gene expression in the mammary gland [95] and therefore eliminates the need for destructive tissue sampling. RNA was electrophoresed through a 1% agarose, low-formaldehyde (1.1%) gel with 1X MOPS [3(N-Morpholino) Propane Sulfonic Acid] buffer at 4°C and then transferred to Zeta-Probe GT Blotting Membrane (BioRad) in 20X SSC (3.0 M sodium chloride, 0.3 M trisodium citrate, pH 7.0) overnight.

Membranes were rinsed in 2X SSC, UV crosslinked at 1200 J (Stratagene UV Stratalinker1800) and hybridized in 25 mL [30% deionised formamide, 5 X SSC, 50 mM sodium acetate, herring sperm DNA (100 μg/μL), 5 mL Denhart’s 50X stock solution, 0.1% SDS] with an [α-^32^P] dCTP-labelled probe [DECAprime II Random Priming DNA Labelling Kit (Ambion)] and incubated for ~16 hr at 42°C. The tammar *ELPRsa*I digested *LGB* (to detect both *LGB* transcripts [96]) and *CST3* probes were either amplified by RT-RCR from tammar mammary gland total RNA or sourced from clones in a tammar mammary gland EST library held by the Cooperative Research Centre for Innovative Dairy Products [19], with plasmid DNA isolated and the cDNA insert amplified by PCR. Membranes were washed (0.1X SSC, 0.1% SDS) twice for 15 min at 60°C, wrapped in cling film, sealed into plastic pockets and exposed to a General Purpose Storage Phosphor screen and scanned on a Typhoon 8600 Scanner (Molecular Dynamics/GE Healthcare). Membranes were stripped of probes by incubation with boiling (100°C) 1X SSC, 0.1% SDS on a shaking platform for two 15 min periods, then rinsed with RT 1X SSC, 0.1% SDS.

### RT-PCR and cloning of *ELP/CTI*

cDNA was generated using Superscript III Reverse Transcriptase (Invitrogen), oligo(dT)20 primer (50 μM; Sigma-Proligo) and 5 μg of total RNA isolated from mammary tissue or cells separated from milk. PCR was performed using 2 μL (10%) of the first strand reaction, the proof-reading Platinum *Taq* DNA Polymerase High Fidelity (Invitrogen), plus the appropriate forward and reverse primers and conditions to amplify *ELP*/*CTI* transcripts (Table 3). PCR products were cloned into the pGEM-T Easy Vector System I (Promega) and sequenced. Full protein-coding *ELP*/*CTI* transcripts were cloned from total RNA extracted from the fat-tailed dunnart, cow and opossum mammary gland tissues and from cells in canine colostrum.

**Table 3:** **Primer sequences and conditions used to amplify*ELP/CTI*genes and transcripts**

**ELP/CTI gene/** **transcript**	**Name**	**Primer Sequence**^**1**^ **5**' **3**'	**PCR Product Size (bp)**	**Primer Conditions**
**FT dunnart transcript**	FT_ELP_F	GTCAAGTGTTATCTACTGGCAGCACCATG	488	94°C for 2 min; 35 cycles of 94°C for 30 sec; 59°C for 30 sec; 68°C for 1 min; 68°C for 10 min
	FT_ELP_R	CCCAAAGTGCTGTTAATGCTTTATTGTAGC		
**Opossum transcript**	mELP_NheI_F	***GCTAGC***AAGGTTTTCTCTCAGTGCCATC	488	94°C for 2 min; 35 cycles of 94°C for 30 sec; 60°C for 30 sec; 68°C for 30 sec; 68°C for 10 min
	mELP_BamHI_R	***GGATCC***TGTTAATGCTTTATTGTACCAG		
**Tammar transcript**	tELP_NheI_F	***GCTAGC***AAGTGTAGTCTACCAGTGGCACC	479	94°C for 2 min; 35 cycles of 94°C for 30 sec; 58°C for 30 sec; 68°C for 30 sec; 68°C for 10 min
	tELP_BamHI_R	***GGATCC***TGTTAATGCTTTATTGTACCAG		
**Dog transcript**	Dog_ELP_Ex1_F	GCCTAGAACATTCAGCTATTGGCACC	449	94°C for 2 min; 35 cycles of 94°C for 30 sec; 55°C for 30 sec; 68°C for 1 min; 68°C for 10 min
	Dog_ELP_Ex3_R	TGAATGTTTTATTGACCTAGACCTGGAGG		
**Cow transcript**	bELP_NheI_F	***GCTAGC***AACTCACAGCTCCTCACACCATG	463	94°C for 2 min; 35 cycles of 94°C for 30 sec; 58°C for 30 sec; 68°C for 30 sec; 68°C for 10 min
	bELP_BamHI_R	***GGATCC***GAACACTTTATTGACCCAGTCCTG		
**FT dunnart gene**	FT_ELP_F	GTCAAGTGTTATCTACTGGCAGCACCATG	4771	94°C for 2 min; 35 cycles of 94°C for 30 sec; 55°C for 30 sec; 68°C for 6 min; 68°C for 10 min
	FT_ELP_R	CCCAAAGTGCTGTTAATGCTTTATTGTAGC		
**Koala gene**	tELP_Ex1_F	GGTAGCAAGTGTAGTCTACCAGTGGCACC	1428	94°C for 2 min; 35 cycles of 94°C for 30 sec; 52°C for 30 sec; 68°C for 4 min; 68°C for 10 min
	tELP_BamHI_R	***GGATCC***TGTTAATGCTTTATTGTACCAG		
**Tammar gene (6.2 kbpromoter)**	T7	TAATACGACTCACTATAGGG	6326	94°C for 2 min; 35 cycles of 94°C for 30 sec; 57°C for 30 sec; 68°C for 8 min; 68°C for 10 min
	tELP_Prom_R	GACTGATCAGACCAATATAAGCTT		
**Tammar gene (7.9 kbpromoter)**	T7	TAATACGACTCACTATAGGG	8044	94°C for 2 min; 35 cycles of 94°C for 30 sec; 57°C for 30 sec; 68°C for 8 min; 68°C for 10 min
	tELP_Ex1_R	GAGGGCCAACGATGGTAAATTTCAT		

^1^Restriction enzyme sites are indicated in bold, italicised text.

### Genomic DNA isolation and cloning

Genomic DNA was isolated from koala and fat-tailed dunnart tissues as described [97]. The *ELP*/*CTI* genes were amplified by PCR (Table 3) using Platinum *Taq* DNA Polymerase and ~200 ng of genomic DNA template, cloned into pGEM-T Easy and sequenced.

### Isolation of the tammar *ELP* gene from a genomic library

A tammar genomic library (liver) in the *E. coli* phage vector lambda EMBL3 T7/SP6 was screened with tammar *ELP* cDNA and a positive clone isolated. The clone was *Sal*I digested and the ~14.7 kb genomic DNA fragment cloned into a modified pBeloBACII plasmid vector. Digestion of pBeloBACII-14.7kbtELP with *Sal*I and *Hind*III yielded three fragments, 6.2 kb *Sal*I/*Hind*III, 5.2 kb *Hind*III/*Hind*III and 3.3 kb *Sal*I/*Hind*III. These fragments were sub-cloned into pBluescript SK and the latter two clones sequenced by the Australian Research Genome Facility (Australia). The remaining 6.2 kb was sequenced (Department of Pathology, The University of Melbourne), providing the full sequence of the genomic clone (14.704 kb). BLAST [98] searches of the NCBI *Macropus eugenii* WGS (Whole Genome Shotgun) trace archives and assembly of hits with CAP3 [99,100] produced a contig of 54,363 bp which included *ELP* and the first 2 exons of *WFDC2*.

### Fluorescence *in situ* hybridisation (FISH)

Metaphase spreads were prepared from the tammar and FISH performed as described [101]. The 14.7 kb tammar *ELP* genomic clone was used as a probe. Slides were examined using a Zeiss Axioplan microscope and images captured using the Spot Advance software package. Pictures were processed with Confocal Assistant, Image J, Adobe Illustrator and Adobe Photoshop. Chromosomal location of *ELP* was verified by at least ten metaphase spreads that had at least three or four signals out of a maximum of four.

### cDNA microarray analysis of tammar *ELP* gene expression

*ELP* gene expression in the tammar mammary gland was investigated by analysing a microarray database [69,102-104] produced from custom-made cDNA microarray slides and total RNA collected from glands at each phase of the lactation cycle [69,102-104]. Glass microarray slides were printed by the Peter MacCallum Cancer Centre Microarray Core Facility, Melbourne, Australia and contained 10,368 tammar cDNA spots which were derived from a commercially prepared (Life Technologies, Rockville, MD, USA), normalised 15,001 tammar mammary gland EST (expressed sequence tag) library. The library was prepared using tammar mammary gland total RNA pooled from various time points in pregnancy (P), lactation (L) and involution (I). These included: day 26P, d55L, d87L, d130L, d180L, d220L, d260L and d5I (tissue from a d45L female 5 days after removal of the pouch young (RPY)) [19]. Gene expression changes in the tammar mammary gland during the reproductive cycle were investigated by a large-scale microarray experiment involving 36 comparisons (72 slides including dye swaps, 144 channels in total) [69,102-104].

Sixteen different time points were used in the experiment: virgin female ~ 300 days old (n = 3), pregnancy (Phase 1: d5P, d25P, d26P; n = 1 per time point), lactation (Phase 2A: d1L, d5L, d80L; Phase 2B: d130L, d168L, d180L; Phase 3: d213L, d220L, d260L; n = 1 per time point) and involution (pouch young were removed at d264L and mammary tissue sampled 1, 5 and 10 days after RPY; n = 1 per time point). Microarray probes were prepared from total RNA (50 μg per sample) using a two-step procedure which involved incorporation of aminoallyl-modified dUTP and then coupling with either Cy3 or Cy5 fluorescent dye [102,104]. Slides were hybridised overnight (14–16 hr) in a humidified chamber [102,104], scanned (Agilent scanner) and the images analysed with Versarray software (Bio-Rad).

Quantile-quantile normalisation within and between microarray slides was implemented using the Limma Package of Bioconductor [105]. The complete data set was analysed simultaneously using a large-scale, linear mixed-model, which included random effects to account for the microarray experiment design, plus gene effects and gene-contrast effects [102,106]. For each time point during pregnancy and lactation, there were a total of 4 different microarray comparisons made; 8 including the Cy3/Cy5 dye swap experiments. For the virgin tissues, there were a total of 12 comparisons, with these values combined for each gene and the average determined. The relative gene expression levels were determined by exponentiation of the gene effects values. The expression levels of the *ELP* and *LGB* milk protein genes and the housekeeping gene *glyceraldehyde 3-phosphate dehydrogenase* (*GAPDH*) were based upon the average expression of n = 3, 7 and 2 non-identical clones on each microarray respectively ± SEM. Microarray experiment data (E-MTAB-1057) was submitted to the EBI Array Express Archive [107].

### Sequence analysis

*ELP/CTI* genes and pseudogenes were identified by BLAST searches of the NCBI GenBank nr and WGS trace archives and BLAST searches of the Ensembl Release 62, April 2011 [49] and UCSC [55] genome databases. We used an Expect-value ≤ 1e-8 as a cut-off for orthologue identification for nucleotide comparisons and gene structure comparison and an E-value ≤ 1e-17 for protein comparisons. Contigs were assembled with CAP3. The following *ELP/CTI* genes and transcripts were submitted to GenBank: the *ELP* gene of the tammar (14.704 kb) [GenBank: JN191335], Southern koala [GenBank: JN191337] and fat-tailed dunnart [GenBank: JN191336], the *ELP* transcripts of the tammar [GenBank: JN191338], fat-tailed dunnart [GenBank: JN191339] and South American opossum [GenBank: JN191340] and *CTI* transcripts of the cow (Holstein-Friesian breed) [GenBank: JN191341] and dog (Labrador breed) [GenBank: JN191342]. Third party annotations of the *ELP*/*CTI* gene were also submitted to GenBank for the cat: [GenBank: BK008083], dog: [GenBank: BK008082], dolphin [GenBank: BK008086], opossum [GenBank: BK008085] and panda [GenBank: BK008084].

The genomic regions encompassing the *PIGTELP/CTI* and *WFDC2* genes in different species were sourced from either the Ensembl or UCSC genome databases for sequence comparisons using mVISTA [64]. These included: dog build *CanFam2* chr24: 35680293–35758485, elephant build *loxAfr3*:

SuperContig_scaffold_19:44809970–44903157, horse build *EquCab2* chr22: 34,465,586-34568786, human build *hg19* chr20: 436717–510935, mouse build *mm9/NCBI37* chr2: 164320020–164401749, opossum build *MonDom5* chr1: 501309327–501453154 and cow build *Btau_4.0* chr13: 74506302–74550554 (included the *PIGT* and *CTI* genes) and 75064658–75139756 (included the *WFDC2* gene). The tammar genome sequences used for comparisons included the incomplete *PIGT* gene in tammar build *Meug_1.0* GeneScaffold_3597: 2268–20682, and a 54,363 bp contig which included tammar *ELP* and the first 2 exons of *WFDC2*. The contig was compiled by BLAST searches of the NCBI *Macropus eugenii* WGS trace archives with the tammar *ELP* gene and assembly with CAP3. The following bovine chromosome 13 genes were also extracted for comparisons: *CTI* (74530701–74533686), *PTI* (75011365–75016221), *STI* (75065067–75069211), *TKDP1* (74843274–74860062), *TKDP2* (74913592–74923363), *TKDP3* (74567402–74577188), *TKDP4* (74874966–74883256), and *TKDP5* (74976879–74983345). The web-based CENSOR tool [108] was used to mask sequences and identify transposable elements by comparison to the Repbase database of repeat elements [66]. Putative exons, transcripts and proteins within genomic sequences were predicted using GENSCAN [109]. However, the third exon of *ELP*/*CTI* was incorrectly predicted by GENSCAN and was therefore determined by manual comparison to known *ELP*/*CTI* splice sites. Splice site location was confirmed by comparison of transcripts and putative proteins. Masked sequences were analysed with mVISTA [64]. Specifications used for each analysis are described in the relevant figure legends.

The ELP/CTI, PTI, STI, SPINT4 (bovine SPINT3 has not been detected) and TKDP family of proteins were subjected to a Prosite database scan [110] to identify putative conserved motifs and post-translational modifications. Putative leader sequences (indicative of secreted proteins) and N-glycosylation sites based upon the NX(S/T) motif were predicted by SignalP 3.0 and NetNGlyc 1.0 Server, respectively, using the Center for Biological Sequence analysis Prediction Servers [56]. Sequences were aligned with CLUSTALW2 [111] and homology within *ELP*/*CTI* transcripts and proteins assessed with MatGAT (Matrix Global Alignment Tool) 2.01 software [112]. MatGAT produces pairwise alignments only and determines homology between each sequence pair based upon the BLOSUM50, BLOSUM62 (used for this study) or PAM250 matrix.

### dN/dS analysis

Selection pressures acting upon different regions of the marsupial ELP and eutherian CTI precursor proteins were determined by dN/dS analysis with MEGA5 software [60]. The protein-coding regions of the marsupial and eutherian transcripts were analysed separately. For each region, the average transition/transversion ratio was calculated using the Maximum Composite Likelihood estimate of the pattern of nucleotide substitution based upon the Tamura-Nei model [113] and then used in the subsequent dN/dS analysis. All codon positions were used, but positions within the alignment containing gaps were eliminated from the analysis. In pairwise comparisons, dN (number of non-synonymous changes per non-synonymous site) and dS (number of synonymous changes per synonymous site) were estimated using the Nei-Gojobori method [114] with modified Jukes-Cantor correction [115] and their variances determined by boostrapping (1000 replications). Codon-based Z-tests for positive (dN > dS), purifying (dN < dS) and neutral (dN = dS) selection were carried out using the Modified Nei-Gojobori method with Jukes-Cantor correction in MEGA5.

### Phylogenetic analysis

The phylogenetic relationship between the protein-coding regions of the marsupial *ELP*, eutherian *CTI*, bovine *TKDP1-5PTI* and *STI* transcripts was investigated using PHYLIP software version 3.69 [116]. Bovine secretory leukocyte protease inhibitor (*SLPI*, GenBank: NM_001098865) was used as an outgroup for the analysis.

Transcripts were aligned with MUSCLE [117] and then 100 bootstrapped alignments generated with SEQBOOT (PHYLIP). The phylogenetic relationship between the sequences was determined using different methods including the character-based maximum likelihood and maximum parsimony methods, as well as distance-based methods. Maximum likelihood trees were generated with DNAMLK which uses a molecular clock assumption. A transition/transversion ratio of 1.34 and a coefficient of variation for the rate of substitution among sites of 0.848 (based upon a gamma distribution with a shape of 1.39) were also specified for the analysis. These values were derived from a Maximum Likelihood test of best fit for 24 different nucleotide substitution models with MEGA5. A Hidden Markov Model using 5 categories, global rearrangements and a randomized input order jumbled once were also used for the DNAMLK analysis. A consensus tree was generated with CONSENSE specifying *SLPI* as an outgroup root, redrawn with RETREE and plotted with DRAWGRAM. Bootstrapped trees were also generated without the molecular clock assumption (DNAML) and using maximum parsimony (DNAPARS). Distance-based analysis on bootstrapped alignments was carried out with DNADIST using the Kimura [118] model of nucleotide substitution. The values used for transition/transversion ratio and gamma distribution were the same as for the maximum likelihood analysis. Trees were generated with the FITCH joining method [119] using global rearrangements, a randomized input order jumbled 10 times and *SLPI* as an outgroup root. The bovine *CTITKDP1-5PTISTISPINT4* and *SLPI* protein-coding transcripts were also analysed with PHYLIP as described above. However, a transition/transversion ratio of 1.39 and a coefficient of variation for the rate of substitution among sites of 0.913 were used.

## Abbreviations

4-DSC, Four disulphide core; aa, Amino acid; AMBP, α1-microglobulin/bikunin precursor; bp, Base pairs; Da, Daltons; EST, Expressed sequence tag; LTR, Long terminal repeat; MER, Medium Reiterated frequency repeat; MYA, Million years ago; nt, Nucleotide; pp, Postpartum; PY, Pouch young; RPY, Removal of pouch young; SPINLW1, Serine peptidase inhibitor-like with Kunitz and WAP domains 1; SPINT, Serine protease inhibitor Kunitz type; TFPI, Tissue factor pathway inhibitor; WAP, Whey acidic protein; WFDC, Wap four disulphide core; WFIKKN, WAP, follistatin/kazal, immunoglobulin, Kunitz and netrin domain containing protein; WGS, Whole genome shotgun.

## Competing interests

The authors declare that they have no competing interests.

## Authors’ contributions

Mammary glands were dissected by KRN and EAP from animals provided by MBR and the Department of Primary Industries, Attwood, Victoria. Experiments were conducted and analysed by EAP, with the exception of the fluorescence *in situ* hybridization of the tammar *ELP* gene, which was performed by AAD, the statistical analysis of microarray experiments which was performed by CML and PCT and the microarray experiments. All authors read, edited and approved the final manuscript.

## Authors’ note

After the submission of this manuscript, we identified the *ELP* gene in the Tasmanian devil (*Sarcophilus harrisii*) by *in silico* analysis of the DEVIL7.0 assembly.

## Supplementary Material

Additional file 1 **Table S1.** Characterisation of the putative functional *ELP*/*CTI* gene, transcript and protein. Click here for file

Additional file 2 **Figure S1** Alignment of the marsupial *ELP* and eutherian *CTI* transcripts. Nucleotide sequences of the tammar [GenBank: JN191338], fat-tailed dunnart (FT) [GenBank: JN191339], opossum [GenBank: JN191340], cow (Holstein-Friesian breed) [GenBank: JN191341], dog (Labrador breed) [GenBank: JN191342] and brushtail possum, plus the transcripts predicted from the *ELP* genes of the stripe-face (SF) dunnart [GenBank: AC186006], koala [GenBank: JN191337], cat [GenBank: BK008083], pig [Ensembl: F1SD34_PIG (ENSSSCT00000008098)], Giant panda [GenBank: BK008084], and Common bottlenose dolphin [GenBank: BK008086] were aligned with ClustalW2. Black shading indicate nucleotide residues common to at least 10 of the species and grey, those that differ. Teal shading indicates nucleotides common to marsupials only, whilst those common to eutherians are shaded blue. The putative translation start site (ATG) is shaded green, the predicted stop codons red, and the polyadenylation signal (AATAAA) is indicated by red text. Nucleotides which encode the signal peptide are indicated by a blue arrow and the Kunitz domain motifs (BPTI KUNITZ 2, Prosite: PS50279 and BPTI KUNITZ 1, Prosite: PS00280) are indicated by red and green lines, respectively. The codons which encode the 6 cysteine residues that form the 3 disulphide bonds of the 51 amino acid Kunitz domain are boxed red. The putative P_1_-P_1_' reactive site residues are shaded yellow and purple respectively. Black arrows indicate the location of *ELP* exons and gaps within the alignment are indicated (−). Click here for file

Additional file 3 **Table S2** Percentage similarity between and within the marsupial *ELP* and eutherian *CTI* transcripts. Pairwise similarities were determined using MatGAT2.01 software [112] based upon alignment of sequence pairs using the BLOSUM62 matrix. **A.***ELP*/*CTI* transcripts (translation start, ATG, to the polyadenylation signal, AATAAA inclusive), **B.***ELP*/*CTI* transcripts (translation start, ATG, to the stop codon inclusive), **C.** Marsupial *ELP* 3'-UTR (untranslated region). Click here for file

Additional file 4 **Table S3** Percentage similarity between and within the marsupial ELP and eutherian CTI peptides. Pairwise similarities were determined using MatGAT2.01 software [112]. **A.** ELP/CTI signal peptide, **B.** ELP/CTI mature peptide, **C.** ELP/CTI N-terminus, **D.** ELP/CTI Kunitz domain motif 2 (51 amino acids), **E.** ELP/CTI Kunitz domain motif 1 (19 amino acids) and **F.** ELP/CTI C-terminus. Click here for file

Additional file 5 **Figure S2** Exon 1 and 2 mutations within selected putative eutherian *CTI* pseudogenes. **A (i).** ClustalW2 alignment of *CTI* exon 1 of the sloth, elephant, human and horse compared to dog exon 1 revealed different putative mutations and deletions. For sloth and horse *CTI* there was a point mutation within the putative translation start site (nt 1–3, methionine codon, ATG). However, human *CTI* exon 1 was disrupted by the deletion of 2 nucleotides (nt 26–27) which would produce a frame-shift (A (ii)). The predicted GT splice site (nt 77–78, orange box) was also disrupted for both the elephant and horse *CTI* sequences. Interestingly, the mutation in the elephant GT splice site would produce a putative protein-coding open reading frame of (279 bp). If this region was transcribed and translated, a precursor protein of 92 amino acids would be secreted. Furthermore, SignalP analysis suggested a mature secreted protein of 70 residues would be produced (data not shown). Nucleotides common to at least four species are boxed black and the remainder, grey. **A (ii).** ClustalW2 alignment of the translated exon 1 region of the functional canine CTI protein revealed mutations in the methionine codon (translation start site) for horse and sloth CTI. In addition, the predicted deletion of 2 nucleotides in human *CTI* would produce a frame-shift. This is shown by the +1 and +2 reading frames of human CTI (2 yellow boxes). Amino acid residues identical to those of the dog are shaded black, with similar amino acid types in grey. **B (i).** ClustalW2 alignment of the functional canine CTI exon 2 with those of the horse, mouse and rat revealed multiple deletions within rodent *CTI*, but a single nucleotide deletion (nt 186) in equine *CTI*. Putative splice sites are indicated (orange boxes). Mutations were also present in the rodent intron 1 AG splice site (nt 20–21), whilst the GT splice site was intact (nt 243–244). The location of the BPTI KUNITZ 1 and 2 motifs within canine *CTI* exon 2 are indicated by green and reds bars respectively. Nucleotides that encode the cysteine residues of the Kunitz domain are shaded red. Mutations in the cysteine encoding nucleotides of C1 were detected for the rat and mouse and also in C2 of murine *CTI*. Nucleotides common to at least four species are shaded black and the fifth grey. **B (ii).** ClustalW2 alignment of the protein encoded by the functional dog *CTI* exon 2 with the putative horse, mouse, and rat proteins revealed multiple mutations and frame-shifts. Equine *CTI* was disrupted by a frame-shift, as shown by alignment of the +2 and +3 reading frames (2 yellow boxes). In contrast, rat CTI has been disrupted by multiple deletions, as evident from the comparison of the +1, +2 and +3 reading frames. This was also true for murine CTI (+1 and +2 reading frames only shown). Gaps have been added to the dog CTI sequence to assist with the alignment (.). Amino acid residues identical to those of the dog are shaded black, with similar residue types shaded grey. Click here for file

Additional file 6 **Figure S3**Transposable elements and simple repeats located within the *PIGT* and *ELP*/*CTI* genes and flanking regions. Conserved transposable elements in the region containing the *PIGT* and *ELP*/*CTI* genes of the opossum, tammar, dog, horse, human, elephant and cow were identified using CENSOR [66,108]. The horizontal axis indicates the relative sizes of the regions compared. Green and red arrows indicate the *PIGT* and *ELP*/*CTI* genes respectively, whilst red arrows with diagonal white stripes indicate the putative horse, human and elephant *CTI* pseudogenes. Exons are indicated by red rectangles. There was a gap in the tammar genome assembly between *PIGT* and *ELP* and the last exon of *PIGT* was missing (red dashed rectangle). Coloured rectangles indicate the different retroelement classes: Transposable elements: DNA transposon (maroon), LTR (long terminal repeat) retrotransposons (brown), Endogenous retrovirus (orange), Non-LTR retrotransposons (blue), interspersed repeat (black) and simple repeat (green). White space indicates the absence of retroelements. Solid lines indicate elements conserved between adjacent species as depicted. Dashed lines indicate elements not present in the adjacent species, but that are preserved in others. Conserved elements are shown in coloured text and those that differ are indicated by black text. Selected retroelements are identified. Click here for file

Additional file 7 **Figure S4** Alignment of the bovine CTI, PTI, STI, TKDP1-5 and SPINT4 precursor proteins. ClustalW2 alignment of the bovine CTI [GenBank: JN191341], PTI [GenBank: P00974], STI [GenBank: NP_991355], TKDP1 [GenBank: NP_991345], TKDP2 [GenBank: AF241777], TKDP3 [GenBank: DAA23071], TKDP4 [GenBank: AAF61250], TKDP5 [GenBank: XP_614808] and SPINT4 [GenBank: XP_614808] precursor proteins. Amino acid residues are numbered based upon the translation start of the precursor proteins and indicated on the right hand side of the alignment. The signal peptides were predicted by SignalP and boxed (blue). The region encoded by the Kunitz domain exon is also boxed (red). The six conserved cysteine residues (C1-C6, C2-C4 and C3-C5), which form the three disulphide bonds that produce a globular protein are shaded red. Notably, C2 and C4 are absent from the TKDP3 and TKDP4 proteins [63]. The BPTI KUNITZ 1 and 2 motifs are indicated (green and red bars respectively) and the putative trypsin interaction (TI) site from the KU motif (NCBI cd00109) is shown by orange triangles. The putative P_1_ reactive site is indicated. Bold, italicised asparagine (*N*) residues indicate predicted sites of post-translational N-glycosylation. Only CTI and SPINT4 were predicted to be N-glycosylated within the Kunitz domain. Amino acid residues that overlap splice sites are shown in red text. Conservation between groups of amino acids with strongly similar properties, i.e., scoring > 0.5 in the Gonnet PAM 250 matrix is indicated (:). Conservation between groups of amino acids with weakly similar properties (scoring < 0.5 in the Gonnet PAM 250 matrix) is also noted (.). Gaps within the alignment are indicated (−). Click here for file

Additional file 8 **Figure S5** Relationship between bovine *CTI*, *PTI*, *STI*, *TKDP1-5* and *SPINT4*. The evolutionary history of the protein-coding regions of the bovine *CTI*, *PTI*, *STI*, *SPINT4* and *TKDP1-*5 transcripts was determined by maximum likelihood analysis based upon a molecular clock assumption using PHYLIP. Bovine *SLPI* was used as an outgroup (data not shown). Numbers at branch points indicate confidence levels as determined by bootstrap values (100 replicates). Transcripts were aligned with MUSCLE and bootstrapped values generated with SEQBOOT. Trees were generated with DNAMLK using a transition/transversion ratio of 1.39, a coefficient of variation for the rate of substitution among sites of 0.913, 5 Hidden Markov Model categories, global rearrangements and a randomised input order jumbled once. The protein-coding regions of the following bovine transcripts were used in the analysis: *CTI* [GenBank: JN191341], *PTI* [GenBank: NM_001001554], *STI* [GenBank: NM_205786], *TKDP1* [GenBank: NM_205776], *TKDP2* [GenBank: NM_001012683], *TKDP3* [GenBank: XM_584746], *TKDP4* [GenBank: NM_205775], *TKDP5* [GenBank: XM_614808], *SPINT4* [Ensembl: ENSBTAT00000039210] and *SLPI* [GenBank: NM_001098865]. Click here for file

Additional file 9 **Figure S6** Genomic arrangement and mVISTA plot of pairwise alignments for the bovine *CTI*, *PTI*, *STI* and *TKDP1-5* genes. **A.** Arrangement and orientation of the bovine chromosome 13 *CTI*, *PTI*, *STI*, *TKDP1 TKDP2*, *TKDP3*, *TKDP4* and *TKDP5* genes. **B. (i-viii)** Homology between the *CTI PTI*, *STI* and *TKDP1-5* genes as determined by mVISTA pairwise sequence alignment. Grey horizontal arrows indicate genes, coding exons are indicated by blue boxes and UTRs of the gene as light green rectangles. The right axis indicates the percentage identity for each pairwise comparison within a 100 bp window, ranging from 10% to 100%. Regions sharing greater than 25% identity are shaded and the black horizontal line indicates 70% identity. The horizontal axis indicates the size of the reference sequence used for each comparison: **(i)** Bovine *CTI*, **(ii)***PTI*, **(iii)***STI*, **(iv)***TKDP1*, **(v)***TKDP2*, **(vi)***TKDP3*, **(vii)***TKDP4*, and **(viii)***TKDP5.* The *CTI* Kunitz domain was most similar to that of *PTI*, *STI*, and *TKDP3*, whilst *PTI* and *STI* homology was greatest within the *TKDP* gene family. Click here for file

Additional file 10 **Figure S7** Transposable elements located within the bovine *CTIPTISTI* and *TKDP1-5* genes. Conserved transposable elements within the *CTIPTISTITKDP1TKDP2TKDP3TKDP4* and *TKDP5* genes (translation start to polyadenylation site, inclusive) were identified using CENSOR [66,108]. The *TKDP* N-domain-encoding exons located between exon 1 (signal- and pro-peptide) and the Kunitz domain-encoding exon (light blue rectangle) were most likely to have arisen due to the “exonisation” of an intron [50]. Their evolutionary history including phylogenetic and dN/dS analysis is discussed in detail [50,51,63]. The second exon of *TKDP5* (A) which is adjacent to a MER21 element may be the ancestral exon of the unique 3-exon N-domains. The ancestral *TKDP5* gene was probably then duplicated to produce either the ancestral *TKDP4TKDP3* or *TKDP2* gene. Within this copied gene, the retroelement (and the exonised intron) was most likely duplicated a further two times (B) and (C), producing a tripartite N-domain of 3 exons: C, B and A (yellow bar 1). This gene subsequently underwent 3 rounds of duplication, resulting in four genes with one N-domain, i.e. three exons which encode the N-domain. Three of these genes, *TKDP4TKDP3* and *TKDP2*, retained the original N-domain. However, for the fourth (*TKDP1*) the tripartite N-domain was replicated twice (yellow bar 2 and yellow bar 3). The horizontal axis indicates the relative sizes of the regions compared, with all genes transcribed from left to right. Exons are indicated by red rectangles, with the exception of the Kunitz domain-encoding exon which is shown as a blue rectangle. Coloured rectangles indicate the different retroelement classes: Transposable elements: DNA transposon (maroon), LTR (long terminal repeat) retrotransposons (brown), Endogenous retrovirus (orange), Non-LTR retrotransposons (blue), interspersed repeat (black) and simple repeat (green). White space indicates the absence of transposable elements. Coloured lines link elements conserved between genes. Selected retroelements are identified, with the conserved fragment size and orientation shown (d = direct, c = complementary). Conserved elements are indicated by coloured text and those that differ, by black text. Arrows indicate the relative orientation of each gene on bovine chromosome 13. Click here for file

Additional file 11 **Table S4** Location, identity and orientation of transposable elements within the bovine *CTIPTISTI* and *TKDP1-5* genes. CENSOR [66,108] output tables showing the predicted identity, location and orientation of retroelement fragments within the bovine *CTIPTISTITKDP1TKDP2TKDP3TKDP4* and *TKDP5* genes. Click here for file

Additional file 12 **Table S5** Tammar wallaby mammary gland cDNA microarray data presented in Figure 7. Global normalised Cy3/Cy5 gene expression data for (i) *ELP*, (ii) *LGB* and (iii) *GAPDH* - throughout the lactation cycle derived from custom-made tammar mammary gland cDNA microarrays. Stages of the reproductive cycle investigated included the virgin female mammary gland, Phase 1 (pregnancy), Phase 2A (early lactation), Phase 2B (mid-lactation) and Phase 3 (late lactation). The EBI ArrayExpress Accesion number (E-MTAB-1057) and GenBank Accession numbers for each clone (microarray spot) are provided, plus the average expression of *ELP*, *CTI* and *LGB* and the associated standard deviation and standard errors. Click here for file
