# Supplementary material for: The mammary gland-specific marsupial ELP and eutherian CTI share a common ancestral gene
Source: BMC Evol Biol. 2012 Jun 8;12:80. doi: 10.1186/1471-2148-12-80 (PMC3426482; doi:10.1186/1471-2148-12-80)
Supplement: Additional file 1 — Table S1. Characterisation of the putative functional ELP/CTI gene, transcript and protein. [file 1471-2148-12-80-S1.pdf]

Additional file 1 - Table S1. Characterisation of the putative functional *ELP/CTI* gene, transcript and protein

Gene	Species	Gene (*in vitro*)	Gene (*in silico* only)	Transcript (*in vitro*)	Transcript (*in silico* only)	Protein (milk/ colostrum)	References
**ELP**	Fat-tailed dunnart	✓		✓	✓		[this study, 27]
	Stripe-faced dunnart	✓			✓		[this study, 27]
	koala	✓			✓		[this study]
	Opossum		✓	✓	✓		[this study, 27]
	Possum	✓		✓	✓	✓	[this study, 25, 28]
	Tammar wallaby	✓		✓		✓	[this study, 11, 13, 20, 21, 26]
**CTI**	Cat		✓		✓		[this study]
	Cow		✓	✓		✓	[this study, 31, 36, 37]
	Dolphin		✓		✓		[this study]
	Dog		✓	✓			[this study]
	Panda		✓		✓		[this study]
	Pig		✓		✓		[this study]
