# Supplementary figures and images for: The mammary gland-specific marsupial ELP and eutherian CTI share a common ancestral gene
Source: BMC Evol Biol. 2012 Jun 8;12:80. doi: 10.1186/1471-2148-12-80 (PMC3426482; doi:10.1186/1471-2148-12-80)

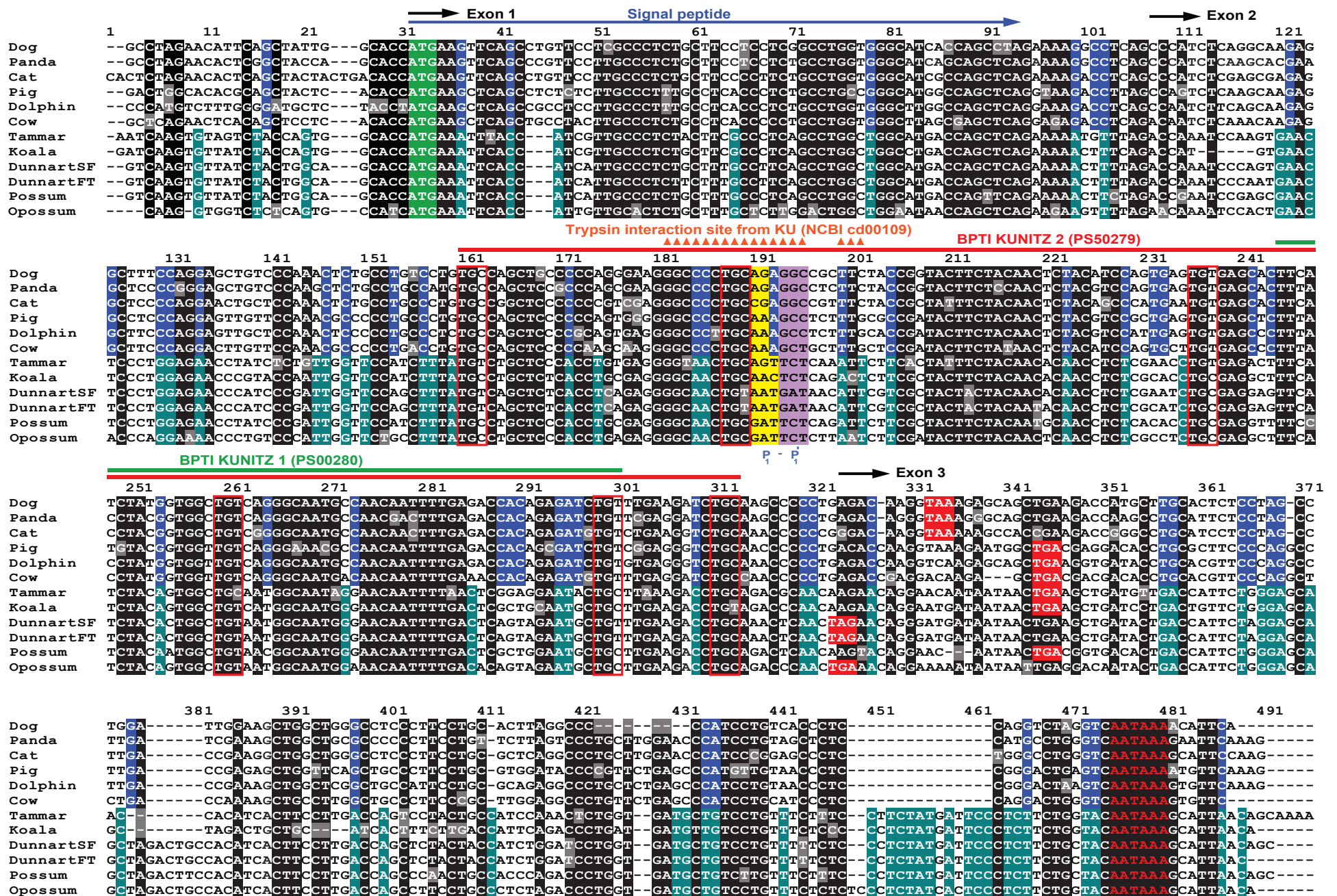

Additional file 2 - Figure S1. Alignment of the marsupial *ELP* and eutherian *CTI* transcripts

Supplement: Additional file 2 — Figure S1 Alignment of the marsupial ELP and eutherian CTI transcripts. Nucleotide sequences of the tammar [GenBank: JN191338], fat-tailed dunnart (FT) [GenBank: JN191339], opossum [GenBank: JN191340], cow (Holstein-Friesian breed) [GenBank: JN191341], dog (Labrador breed) [GenBank: JN191342] and brushtail possum, plus the transcripts predicted from the ELP genes of the stripe-face (SF) dunnart [GenBank: AC186006], koala [GenBank: JN191337], cat [GenBank: BK008083], pig [Ensembl: F1SD34_PIG (ENSSSCT00000008098)], Giant panda [GenBank: BK008084], and Common bottlenose dolphin [GenBank: BK008086] were aligned with ClustalW2. Black shading indicate nucleotide residues common to at least 10 of the species and grey, those that differ. Teal shading indicates nucleotides common to marsupials only, whilst those common to eutherians are shaded blue. The putative translation start site (ATG) is shaded green, the predicted stop codons red, and the polyadenylation signal (AATAAA) is indicated by red text. Nucleotides which encode the signal peptide are indicated by a blue arrow and the Kunitz domain motifs (BPTI KUNITZ 2, Prosite: PS50279 and BPTI KUNITZ 1, Prosite: PS00280) are indicated by red and green lines, respectively. The codons which encode the 6 cysteine residues that form the 3 disulphide bonds of the 51 amino acid Kunitz domain are boxed red. The putative P1-P1' reactive site residues are shaded yellow and purple respectively. Black arrows indicate the location of ELP exons and gaps within the alignment are indicated (−). [file 1471-2148-12-80-S2.pdf]
