# Supplementary material for: The mammary gland-specific marsupial ELP and eutherian CTI share a common ancestral gene
Source: BMC Evol Biol. 2012 Jun 8;12:80. doi: 10.1186/1471-2148-12-80 (PMC3426482; doi:10.1186/1471-2148-12-80)
Supplement: Additional file 3 — Table S2 Percentage similarity between and within the marsupial ELP and eutherian CTI transcripts. Pairwise similarities were determined using MatGAT2.01 software [112] based upon alignment of sequence pairs using the BLOSUM62 matrix. A.ELP/CTI transcripts (translation start, ATG, to the polyadenylation signal, AATAAA inclusive), B.ELP/CTI transcripts (translation start, ATG, to the stop codon inclusive), C. Marsupial ELP 3'-UTR (untranslated region). [file 1471-2148-12-80-S3.pdf]

Additional file 3 - Table S2. Percentage similarity between and within the marsupial *ELP* and eutherian *CTI* transcripts

Pairwise similarities were calculated with MatGat version 2.01 using the BLOSUM62 matrix.
2A. ELP/CTI transcripts (ATG to AATAAA, inclusive)**, 2B.** ELP/CTI transcripts (ATG to stop codon, inclusive)**,**
2C. Marsupial ELP 3'UTR

**2A. *ELP/CTI Transcripts*[1]**	**1**	**2**	**3**	**4**	**5**	**6**	**7**	**8**	**9**	**10**	**11**
**1. Tammar**											
**2. DunnartSF**	83.5										
**3. DunnartFT**	82.8	99.1									
**4. Koala**	83.4	83.5	83.1								
**5. Opossum**	79.1	82.9	82.7	80.7							
**6. Possum**	85.9	86.9	86.9	85.7	82.5						
**7. Cat**	58.6	60.3	59.4	61.7	59.8	62.0					
**8. Cow**	56.8	58.2	57.8	61.2	60.7	58.9	75.6				
**9. Dog**	58.9	58.9	57.3	62.6	62.0	58.0	82.4	76.7			
**10. Dolphin**	56.8	59.4	59.1	60.3	58.2	58.9	79.7	86.0	79.4		
**11. Panda**	58.2	59.8	59.4	62.2	63.6	59.8	84.3	76.1	86.6	79.4	
**12. Pig**	56.1	57.6	57.6	61.4	58.9	57.5	75.2	81.5	75.0	86.7	75.5

[1] ATG to AATAAA, inclusive

**2B. *ELP/CTI Transcripts*[2]**	**1**	**2**	**3**	**4**	**5**	**6**	**7**	**8**	**9**	**10**	**11**
**1. Tammar**											
**2. DunnartSF**	78.5										
**3. DunnartFT**	77.6	98.6									
**4. Koala**	85.6	80.7	80.1								
**5. Opossum**	74.0	81.4	81.1	78.4							
**6. Possum**	86.2	82.2	82.2	89.3	78.0						
**7. Cat**	59.6	61.4	61.4	63.4	61.7	65.4					
**8. Cow**	59.0	56.1	56.1	62.8	61.2	60.9	75.6				
**9. Dog**	60.6	59.7	59.7	63.1	64.4	60.5	86.5	78.2			
**10. Dolphin**	59.7	57.5	57.5	61.9	57.5	60.6	79.0	87.3	79.4		
**11. Panda**	58.7	60.7	60.7	64.1	64.4	62.1	85.1	75.6	90.4	78.7	
**12. Pig**	58.1	57.1	57.1	63.8	59.0	59.7	76.5	82.2	76.8	88.3	76.2

[2] ATG to stop codon, inclusive

**2C. Marsupial *ELP* 3'UTR**	**1**	**2**	**3**	**4**	**5**
**1. Tammar**					
**2. DunnartSF**	88.5				
**3. DunnartFT**	88.5	100.0			
**4. Koala**	88.5	88.5	88.5		
**5. Opossum**	87.0	87.0	87.0	87.0	
**6. Possum**	92.3	92.3	92.3	88.5	87.0
