# Supplementary material for: The mammary gland-specific marsupial ELP and eutherian CTI share a common ancestral gene
Source: BMC Evol Biol. 2012 Jun 8;12:80. doi: 10.1186/1471-2148-12-80 (PMC3426482; doi:10.1186/1471-2148-12-80)
Supplement: Additional file 4 — Table S3 Percentage similarity between and within the marsupial ELP and eutherian CTI peptides. Pairwise similarities were determined using MatGAT2.01 software [112]. A. ELP/CTI signal peptide, B. ELP/CTI mature peptide, C. ELP/CTI N-terminus, D. ELP/CTI Kunitz domain motif 2 (51 amino acids), E. ELP/CTI Kunitz domain motif 1 (19 amino acids) and F. ELP/CTI C-terminus. [file 1471-2148-12-80-S4.pdf]

Additional file 4 - Table S3. Percentage similarity between and within the marsupial ELP and eutherian CTI peptides

3A. ELP/CTI mature peptide, **3B.**ELP/CTI signal peptide, **3C.** ELP/CTI N-terminus, **3D.** ELP/CTI Kunitz domain motif 2 (51 amino acids), **3E.** ELP/CTI Kunitz domain motif 1 (19 amino acids) and **3F.** ELP/CTI C-terminus.

3A. ELP/CTI Mature Peptide	1	2	3	4	5	6	7	8	9	10	11
1. Tammar											
2. DunnartSF	71.1										
3. DunnartFT	71.1	100.0									
4. Koala	79.5	76.5	76.5								
5. Opossum	67.5	81.6	81.6	76.5							
6. Possum	80.7	75.6	75.6	81.7	72.0						
7. Cat	54.2	54.4	54.4	59.3	50.6	54.9					
8. Cow	56.6	53.7	53.7	62.2	51.2	61.0	73.2				
9. Dog	56.6	57.0	57.0	60.5	55.7	61.0	88.6	74.4			
10. Dolphin	56.6	53.0	53.0	57.8	48.2	60.2	81.9	86.7	78.3		
11. Panda	49.4	53.2	53.2	58.0	46.8	51.2	84.8	70.7	86.1	73.5	
12. Pig	53.0	53.0	53.0	51.8	44.6	55.4	73.5	80.7	73.5	85.5	68.7

3B. ELP/CTI Signal Peptide	1	2	3	4	5	6	7	8	9	10	11
1. Tammar											
2. DunnartSF	90.0										
3. DunnartFT	95.0	95.0									
4. Koala	95.0	95.0	90.0								
5. Opossum	90.0	90.0	85.0	95.0							
6. Possum	85.0	85.0	80.0	90.0	85.0						
7. Cat	66.7	76.2	71.4	71.4	71.4	71.4					
8. Cow	57.1	61.9	57.1	61.9	61.9	71.4	71.4				
9. Dog	71.4	71.4	66.7	76.2	81.0	76.2	76.2	57.1			
10. Dolphin	61.9	61.9	57.1	66.7	66.7	76.2	71.4	90.5	61.9		
11. Panda	71.4	71.4	66.7	76.2	76.2	76.2	85.7	71.4	85.7	76.2	
12. Pig	61.9	61.9	57.1	66.7	66.7	71.4	71.4	81.0	61.9	85.7	71.4

3C. ELP/CTI N-terminus	1	2	3	4	5	6	7	8	9	10	11
1. Tammar											
2. DunnartSF	81.8										
3. DunnartFT	81.8	100.0									
4. Koala	63.6	77.3	77.3								
5. Opossum	59.1	72.7	72.7	59.1							
6. Possum	86.4	86.4	86.4	72.7	59.1						
7. Cat	50.0	45.5	45.5	59.1	27.3	50.0					
8. Cow	45.5	40.9	40.9	54.5	22.7	50.0	72.7				
9. Dog	50.0	45.5	45.5	50.0	36.4	54.5	77.3	68.2			
10. Dolphin	40.9	36.4	36.4	45.5	18.2	45.5	86.4	86.4	72.7		
11. Panda	50.0	45.5	45.5	59.1	18.2	54.5	81.8	59.1	81.8	68.2	
12. Pig	45.5	40.9	40.9	40.9	18.2	50.0	72.7	86.4	63.6	90.9	59.1

3D. ELP/CTI Kunitz Domain motif 2 (51 amino acids)	1	2	3	4	5	6	7	8	9	10	11
1. Tammar											
2. DunnartSF	76.5										
3. DunnartFT	76.5	100.0									
4. Koala	84.3	84.3	84.3								
5. Opossum	80.4	86.3	86.3	90.2							
6. Possum	80.4	78.4	78.4	86.3	86.3						
7. Cat	62.7	62.7	62.7	62.7	62.7	64.7					
8. Cow	64.7	64.7	64.7	66.7	66.7	68.6	78.4				
9. Dog	64.7	66.7	66.7	66.7	66.7	68.6	94.1	80.4			
10. Dolphin	64.7	66.7	66.7	62.7	64.7	68.6	86.3	88.2	84.3		
11. Panda	54.9	60.8	60.8	60.8	60.8	56.9	88.2	78.4	88.2	80.4	
12. Pig	62.7	66.7	66.7	60.8	62.7	64.7	80.4	80.4	82.4	84.3	76.5

3E. ELP/CTI Kunitz Domain motif 1 (19 amino acids)	1	2	3	4	5	6	7	8	9	10	11
1. Tammar											
2. DunnartSF	84.2										
3. DunnartFT	84.2	100.0									
4. Koala	84.2	100.0	100.0								
5. Opossum	84.2	100.0	100.0	100.0							
6. Possum	84.2	100.0	100.0	100.0	100.0						
7. Cat	63.2	68.4	68.4	68.4	68.4	68.4					
8. Cow	63.2	68.4	68.4	68.4	68.4	68.4	94.7				
9. Dog	68.4	73.7	73.7	73.7	73.7	73.7	94.7	89.5			
10. Dolphin	63.2	68.4	68.4	68.4	68.4	68.4	100.0	94.7	94.7		
11. Panda	63.2	68.4	68.4	68.4	68.4	68.4	100.0	94.7	94.7	100.0	
12. Pig	63.2	68.4	68.4	68.4	68.4	68.4	89.5	84.2	94.7	89.5	89.5

3F. ELP/CTI C-terminus	1	2	3	4	5	6	7	8	9	10	11
1. Tammar											
2. DunnartSF	20.0										
3. DunnartFT	20.0	100.0									
4. Koala	90.0	20.0	20.0								
5. Opossum	20.0	66.7	66.7	30.0							
6. Possum	70.0	33.3	33.3	70.0	22.2						
7. Cat	20.0	16.7	16.7	30.0	33.3	22.2					
8. Cow	40.0	22.2	22.2	50.0	33.3	44.4	44.4				
9. Dog	30.0	16.7	16.7	40.0	33.3	33.3	83.3	55.6			
10. Dolphin	50.0	20.0	20.0	60.0	30.0	50.0	50.0	80.0	60.0		
11. Panda	20.0	16.7	16.7	30.0	33.3	11.1	66.7	55.6	83.3	50.0	
12. Pig	20.0	10.0	10.0	30.0	10.0	20.0	40.0	70.0	50.0	80.0	50.0
