# Supplementary material for: The mammary gland-specific marsupial ELP and eutherian CTI share a common ancestral gene
Source: BMC Evol Biol. 2012 Jun 8;12:80. doi: 10.1186/1471-2148-12-80 (PMC3426482; doi:10.1186/1471-2148-12-80)
Supplement: Additional file 5 — Figure S2 Exon 1 and 2 mutations within selected putative eutherian CTI pseudogenes. A (i). ClustalW2 alignment of CTI exon 1 of the sloth, elephant, human and horse compared to dog exon 1 revealed different putative mutations and deletions. For sloth and horse CTI there was a point mutation within the putative translation start site (nt 1–3, methionine codon, ATG). However, human CTI exon 1 was disrupted by the deletion of 2 nucleotides (nt 26–27) which would produce a frame-shift (A (ii)). The predicted GT splice site (nt 77–78, orange box) was also disrupted for both the elephant and horse CTI sequences. Interestingly, the mutation in the elephant GT splice site would produce a putative protein-coding open reading frame of (279 bp). If this region was transcribed and translated, a precursor protein of 92 amino acids would be secreted. Furthermore, SignalP analysis suggested a mature secreted protein of 70 residues would be produced (data not shown). Nucleotides common to at least four species are boxed black and the remainder, grey. A (ii). ClustalW2 alignment of the translated exon 1 region of the functional canine CTI protein revealed mutations in the methionine codon (translation start site) for horse and sloth CTI. In addition, the predicted deletion of 2 nucleotides in human CTI would produce a frame-shift. This is shown by the +1 and +2 reading frames of human CTI (2 yellow boxes). Amino acid residues identical to those of the dog are shaded black, with similar amino acid types in grey. B (i). ClustalW2 alignment of the functional canine CTI exon 2 with those of the horse, mouse and rat revealed multiple deletions within rodent CTI, but a single nucleotide deletion (nt 186) in equine CTI. Putative splice sites are indicated (orange boxes). Mutations were also present in the rodent intron 1 AG splice site (nt 20–21), whilst the GT splice site was intact (nt 243–244). The location of the BPTI KUNITZ 1 and 2 motifs within canine CTI exon 2 are indicated by gre [file 1471-2148-12-80-S5.pdf]

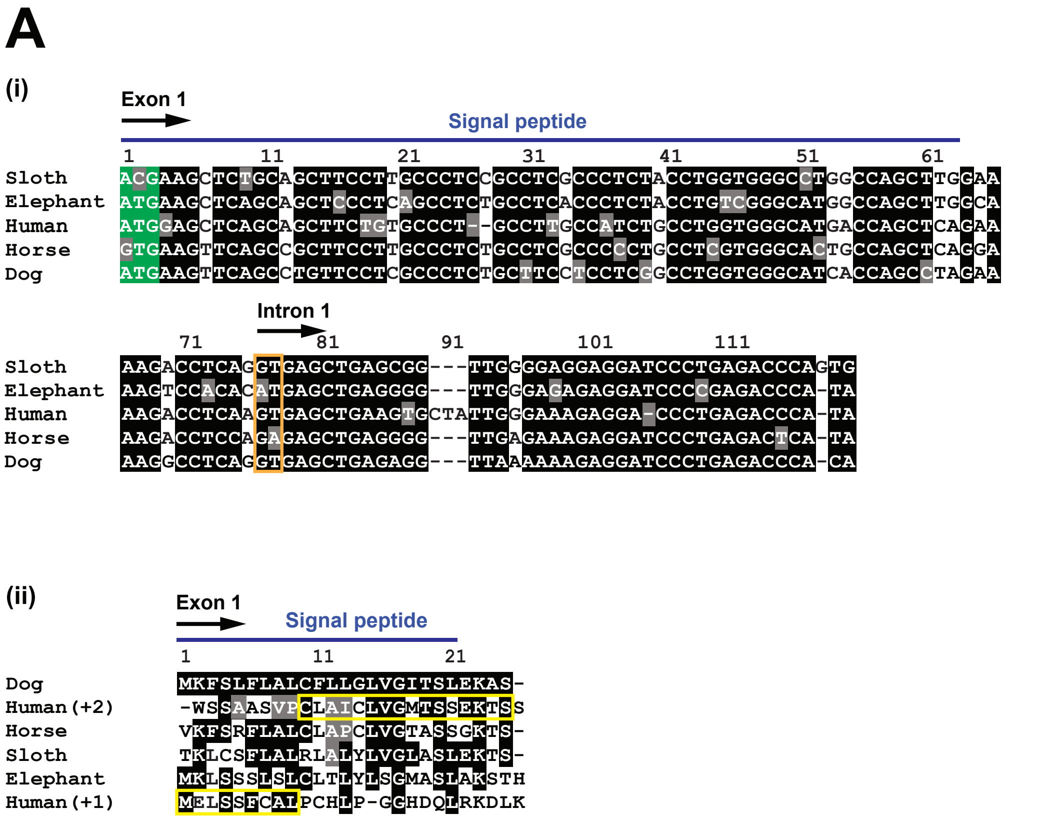

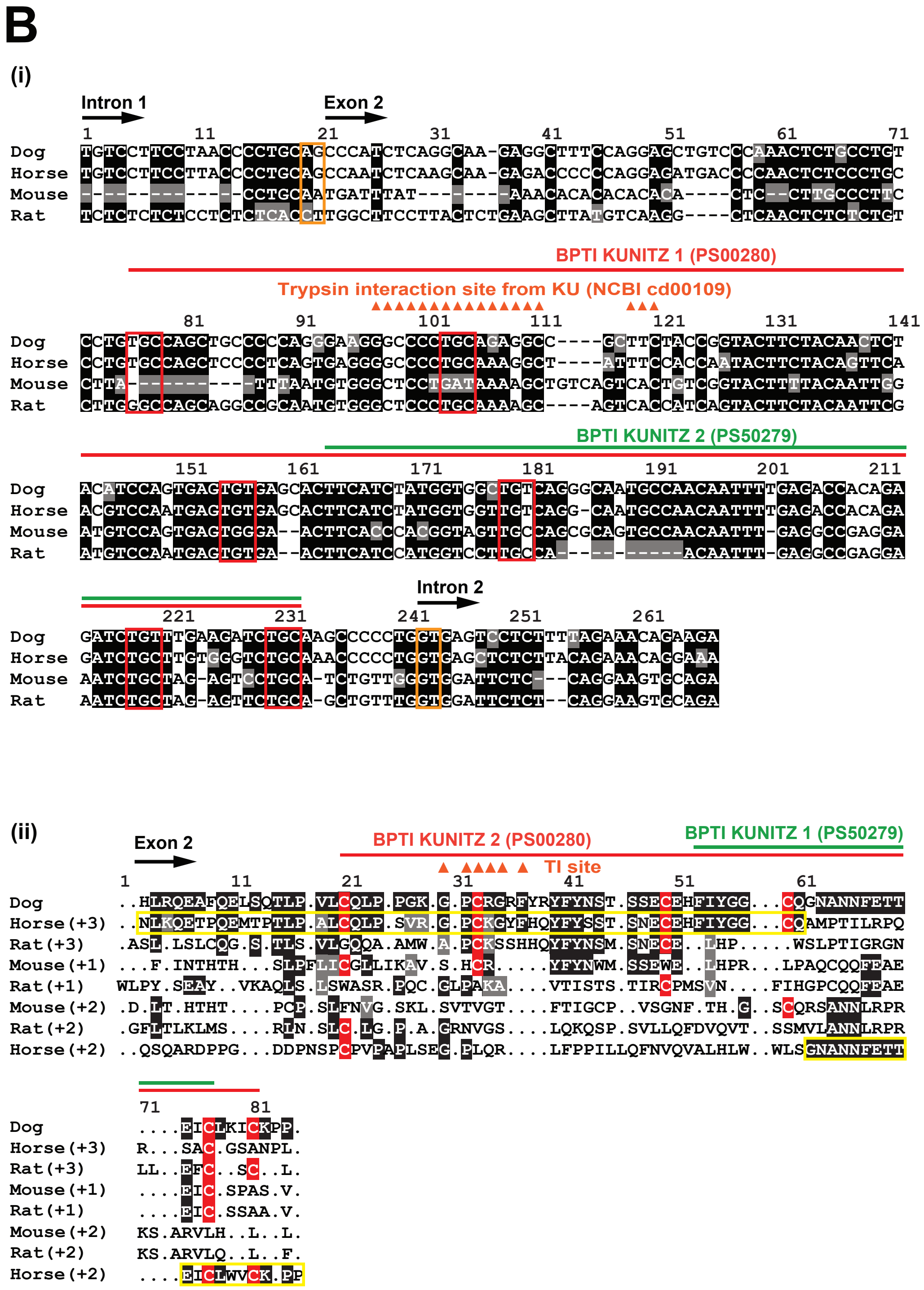

Additional file 5 - Figure S2. Exon 1 and 2 mutations within selected putative eutherian *CTI* pseudogenes.

A (i). Alignment of the 'functional' canine *CTI* exon 1 with the putative disrupted exon 1 of the sloth, elephant, human and horse.**(ii)**. Alignment of the translated putative CTI exon 1 peptides of the dog, sloth, elephant and human. The +2 and +3 frames of human CTI are included in the alignment to show that deletions within exon 1 would produce a frameshift (yellow boxes).

B (i). Alignment of the functional canine *CTI exon* 2 with the putative disrupted exon 2 of the rat, mouse and horse. The BPTI KUNITZ 1 and 2 motifs are shown in green and red bars respectively. The putative trypsin interaction site from the KU motif (NCBI cd00109) is indicated by orange triangles.

(ii). Alignment of the translated 'functional' canine *CTI* exon 2 region with the equivalent region in the rat, mouse and horse. The +2 and +3 reading frames are shown for equine CTI, the +1 and +2 frames for the mouse and all 3 reading frames for rat CTI. Yellow boxes indicate the putative frame-shift in equine ELP.
