# Supplementary material for: The mammary gland-specific marsupial ELP and eutherian CTI share a common ancestral gene
Source: BMC Evol Biol. 2012 Jun 8;12:80. doi: 10.1186/1471-2148-12-80 (PMC3426482; doi:10.1186/1471-2148-12-80)
Supplement: Additional file 6 — Figure S3Transposable elements and simple repeats located within the PIGT and ELP/CTI genes and flanking regions. Conserved transposable elements in the region containing the PIGT and ELP/CTI genes of the opossum, tammar, dog, horse, human, elephant and cow were identified using CENSOR [66,108]. The horizontal axis indicates the relative sizes of the regions compared. Green and red arrows indicate the PIGT and ELP/CTI genes respectively, whilst red arrows with diagonal white stripes indicate the putative horse, human and elephant CTI pseudogenes. Exons are indicated by red rectangles. There was a gap in the tammar genome assembly between PIGT and ELP and the last exon of PIGT was missing (red dashed rectangle). Coloured rectangles indicate the different retroelement classes: Transposable elements: DNA transposon (maroon), LTR (long terminal repeat) retrotransposons (brown), Endogenous retrovirus (orange), Non-LTR retrotransposons (blue), interspersed repeat (black) and simple repeat (green). White space indicates the absence of retroelements. Solid lines indicate elements conserved between adjacent species as depicted. Dashed lines indicate elements not present in the adjacent species, but that are preserved in others. Conserved elements are shown in coloured text and those that differ are indicated by black text. Selected retroelements are identified. [file 1471-2148-12-80-S6.pdf]

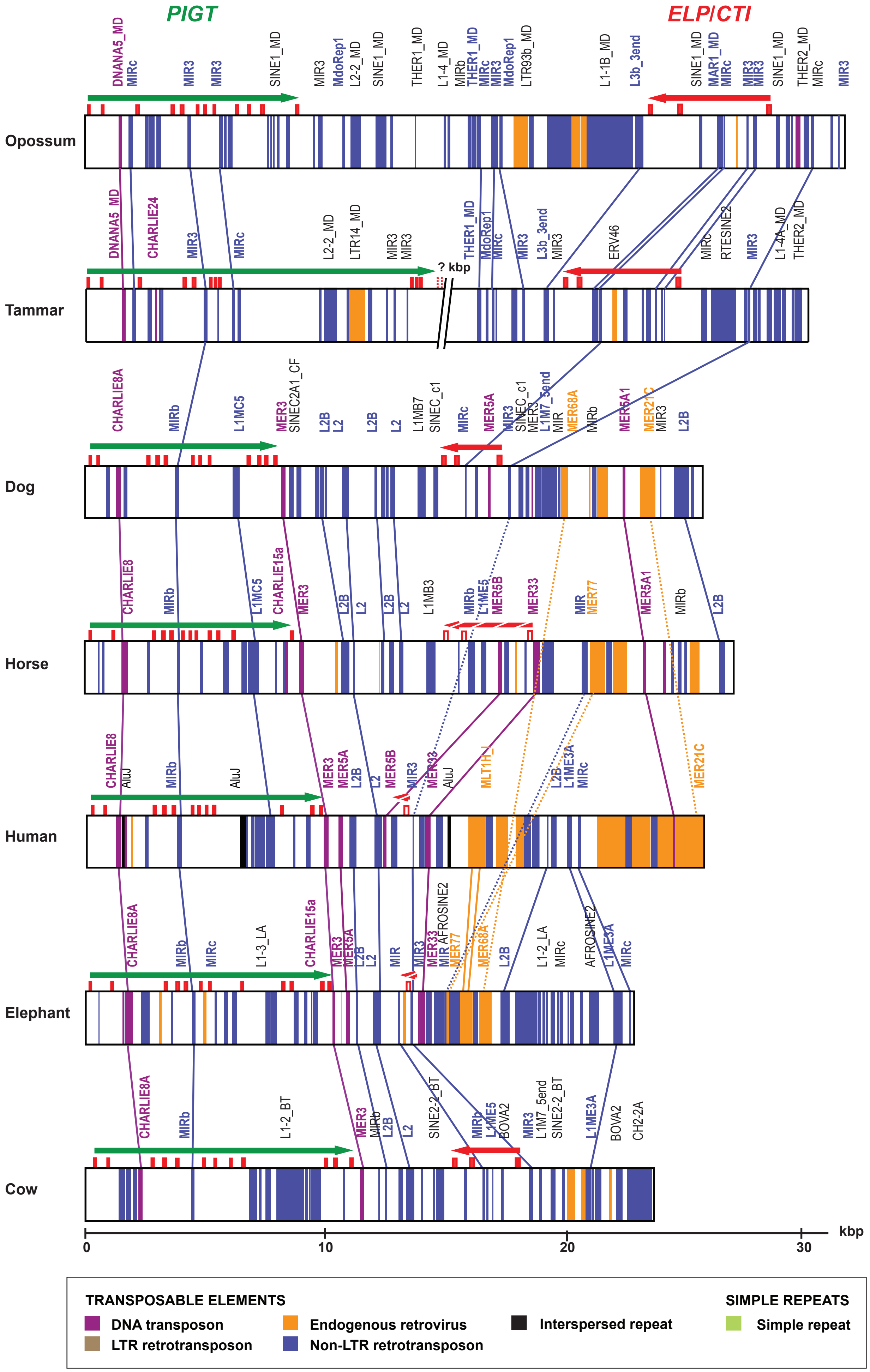

Additional file 6 - Figure S3. Transposable elements and simple repeats located within the *PIGT* and *ELP/CTI* genes and flanking regions
