# Supplementary material for: The mammary gland-specific marsupial ELP and eutherian CTI share a common ancestral gene
Source: BMC Evol Biol. 2012 Jun 8;12:80. doi: 10.1186/1471-2148-12-80 (PMC3426482; doi:10.1186/1471-2148-12-80)
Supplement: Additional file 7 — Figure S4 Alignment of the bovine CTI, PTI, STI, TKDP1-5 and SPINT4 precursor proteins. ClustalW2 alignment of the bovine CTI [GenBank: JN191341], PTI [GenBank: P00974], STI [GenBank: NP_991355], TKDP1 [GenBank: NP_991345], TKDP2 [GenBank: AF241777], TKDP3 [GenBank: DAA23071], TKDP4 [GenBank: AAF61250], TKDP5 [GenBank: XP_614808] and SPINT4 [GenBank: XP_614808] precursor proteins. Amino acid residues are numbered based upon the translation start of the precursor proteins and indicated on the right hand side of the alignment. The signal peptides were predicted by SignalP and boxed (blue). The region encoded by the Kunitz domain exon is also boxed (red). The six conserved cysteine residues (C1-C6, C2-C4 and C3-C5), which form the three disulphide bonds that produce a globular protein are shaded red. Notably, C2 and C4 are absent from the TKDP3 and TKDP4 proteins [63]. The BPTI KUNITZ 1 and 2 motifs are indicated (green and red bars respectively) and the putative trypsin interaction (TI) site from the KU motif (NCBI cd00109) is shown by orange triangles. The putative P1 reactive site is indicated. Bold, italicised asparagine (N) residues indicate predicted sites of post-translational N-glycosylation. Only CTI and SPINT4 were predicted to be N-glycosylated within the Kunitz domain. Amino acid residues that overlap splice sites are shown in red text. Conservation between groups of amino acids with strongly similar properties, i.e., scoring > 0.5 in the Gonnet PAM 250 matrix is indicated (:). Conservation between groups of amino acids with weakly similar properties (scoring < 0.5 in the Gonnet PAM 250 matrix) is also noted (.). Gaps within the alignment are indicated (−). [file 1471-2148-12-80-S7.pdf]

```
                 Signal peptide
TKDP4      --MNRLCLSAALLLLLVILVDSTPVYEHHTQDQGLETSHGRRLEKRSVTDLISAFMDAMV 58
TKDP5      MKMSRLCLSAALLFLPVILVDSTPVYEQNTQDQGLV------------------------ 36
TKDP3      --MNRLCLSAALLFLLVILVDSTLVNIHHIQDEGLETSHRRGPKKHSTKDMIKNIIRGVA 58
TKDP1      --MRQLCLSSALLFLLVILVDSTPLNIHHIQDEGVETSHRRGPEKRSVIDVVTSIIDGVA 58
TKDP2      --MSRLCLSAALLFLLVILVDSTPVYEHHTQDQGLETSHRRGPEKRSIIDVISHVIDGVV 58
PTI        MKMSRLCLSVALLVLLGTLAASTP------------------------------------ 24
STI        MKMSRLCLSIALLVLLGTLAASTP------------------------------------ 24
CTI        --MKLSCLLALCLTPCLVGLASSGE----------------------------------- 23
SPINT4     --MKLIELGFLLGLFTSLLTTPLMG----------------------------------- 23
             *    *             .

TKDP4      IVAELLKG---------------------------------------------------- 66
TKDP5      ------------------------------------------------------------
TKDP3      TGAKIANAGSGF------------------------------------------------ 70
TKDP1      TGTKIVKNGAGLLTGLAEIITKAIKGQVMISRIQFDNHTQEELPTLNIEYSTLSEENKGV 118
TKDP2      KGTKIFHG---------------------------------------------------- 66
PTI        ------------------------------------------------------------
STI        ------------------------------------------------------------
CTI        ------------------------------------------------------------
SPINT4     ------------------------------------------------------------

TKDP4      ------------------------------------------------------------
TKDP5      ------------------------------------------------------------
TKDP3      ------------------------------------------------------------
TKDP1      ETSHRKGPEKRSVIDVVTSIIDGVATGTKIVTKGASILTGLAEIINKAIRGQVMISRIQF 178
TKDP2      ------------------------------------------------------------
PTI        ------------------------------------------------------------
STI        ------------------------------------------------------------
CTI        ------------------------------------------------------------
SPINT4     ------------------------------------------------------------

TKDP4      ------------------------------------------------------------
TKDP5      ------------------------------------------------------------
TKDP3      ------------------------------------------------------------
TKDP1      NNHTLEEFPKLNIEYSTLSEDNTGVETSRRRGPEKRSVIDVVTSIIDGVATGTKIVKKGT 238
TKDP2      ------------------------------------------------------------
PTI        ------------------------------------------------------------
STI        ------------------------------------------------------------
CTI        ------------------------------------------------------------
SPINT4     ------------------------------------------------------------

                                                      Kunitz domain-encoding exon
TKDP4      --VGFDELKGLPQTMSQMMISGRQLENHAVEVFPTQ-----TLREENKADSKPAFCLEPK 119
TKDP5      ------------------MISGKQLEKRSVKELPTA-----VLQEENKATSRPAFCLEHK 73
TKDP3      --IREVVKAMREGNKGHVKTSGAQLKNHTVEESPTFNTQQLILSEKNKADSRPAFCLEPK 128
TKDP1      SILTGLAEIINKAIKGQVMISGIQFNNHTLEEYQTLKIEYSALNEENKAASKPALCLEPK 298
TKDP2      --LKGLADIISNGIQGQVMISGTQLENHTVEEFLTQILKH--------AASKPEFCMEPE 116
PTI        ---------------------GCDTSNQAK-------------------AQRPDFCLEPP 44
STI        ---------------------GCDTSNQAK-------------------AQRPDFCLEPP 44
CTI        --------------------TSDNLKQEASQD---------------LFQTPPDLCQLPQ 48
SPINT4     ---------------------GMTQITDMI------------------CKKFKDRCKMPL 44
                                                                   *
             ▲▲▲▲▲ ▲ TI site         .   BPTI KUNITZ 1 (PS00280)
              BPTI KUNITZ 2 (PS50279)
TKDP4      VTGHSKSSWPRYFYNAETGHCEQFTYGGLGGNKNNFITEEECMKTCGQGAGSLREHAKSG 179
TKDP5      FSGPCITPEIRYFYNAKTGHCEHFIYGGCNGKKNNFLTEEDCIKTCGQGAGSP------- 126
TKDP3      VVGHGTTKMPRYFYDAKTGHCEPFTYGSLGGNKNNFLTIEDCMKTCGQGAGSL------- 181
TKDP1      VTGGCNAVMTRYFYNAQNGLCEQFVYDGCEGNGNNFEKLEDCMKTCSQEAGSL------- 351
TKDP2      LKGPCKDQMTRYFYNAKTRYCEPFVYGGCEGNKNNFQTLSHCIVTCCPVPVTM------- 169
PTI        YTGPCKARMIRYFYNAKAGLCQPFVYGGCRAKRNNFKSSEDCMRTCGGAIGPWENL---- 100
STI        YTGPCKAKMIRYFYNAKAGFCETFVYGGCKAKSNNFRSAEDCMRTCGGAIGPRENL---- 100
CTI        ARGPCKAALLRYFYNSTSSACEPFTYGGCQGNDNNFETTEMCLRICQPPETEDKS----- 103
SPINT4     NFGSCYDIQFRYFYNITSGLCESFVYTGCDGNLNNYRLKIECQMTCEKKYIKER------ 98
             *  P1-P1'  ****:     *: * *  .  .: **:      *   *

TKDP4      PQKP 183
TKDP5      ----
TKDP3      ----
TKDP1      ----
TKDP2      ----
PTI        ----
STI        ----
CTI        ----
SPINT4     ----
```

Additional file 7 - Figure S4. Alignment of the bovine CTI, PTI, STI, TKDP1-5 and SPINT4 precursor proteins
