# Supplementary material for: The mammary gland-specific marsupial ELP and eutherian CTI share a common ancestral gene
Source: BMC Evol Biol. 2012 Jun 8;12:80. doi: 10.1186/1471-2148-12-80 (PMC3426482; doi:10.1186/1471-2148-12-80)
Supplement: Additional file 8 — Figure S5 Relationship between bovine CTI, PTI, STI, TKDP1-5 and SPINT4. The evolutionary history of the protein-coding regions of the bovine CTI, PTI, STI, SPINT4 and TKDP1-5 transcripts was determined by maximum likelihood analysis based upon a molecular clock assumption using PHYLIP. Bovine SLPI was used as an outgroup (data not shown). Numbers at branch points indicate confidence levels as determined by bootstrap values (100 replicates). Transcripts were aligned with MUSCLE and bootstrapped values generated with SEQBOOT. Trees were generated with DNAMLK using a transition/transversion ratio of 1.39, a coefficient of variation for the rate of substitution among sites of 0.913, 5 Hidden Markov Model categories, global rearrangements and a randomised input order jumbled once. The protein-coding regions of the following bovine transcripts were used in the analysis: CTI [GenBank: JN191341], PTI [GenBank: NM_001001554], STI [GenBank: NM_205786], TKDP1 [GenBank: NM_205776], TKDP2 [GenBank: NM_001012683], TKDP3 [GenBank: XM_584746], TKDP4 [GenBank: NM_205775], TKDP5 [GenBank: XM_614808], SPINT4 [Ensembl: ENSBTAT00000039210] and SLPI [GenBank: NM_001098865]. [file 1471-2148-12-80-S8.pdf]

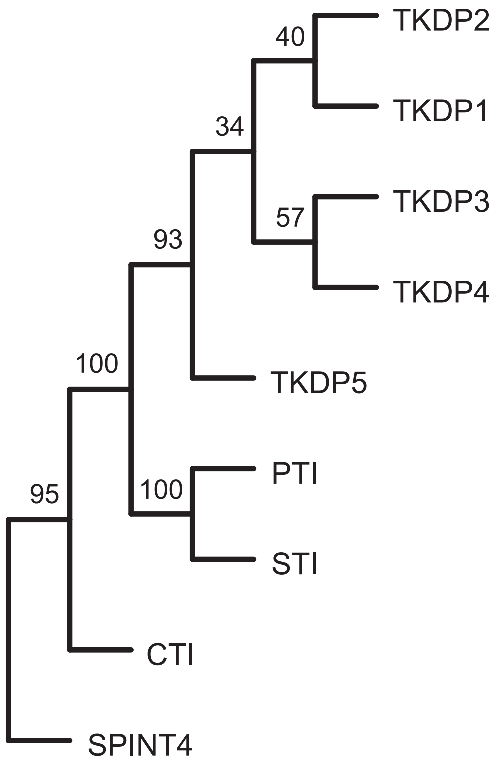

Additional file 8 - Figure S5. Relationship between bovine CTI, PTI, STI, the TKDPs and SPINT4

Phylogenetic relationship between the protein-coding regions of the *CTI*, *PTI*, *STI*, *TKDP1-5* and *SPINT4* transcripts.The tree was constructed using the maximum likelihood model of nucleotide substution based upon the assumption of a molecular clock. The *SLPI* transcript (omitted) was used to root the tree.
