# Supplementary material for: The mammary gland-specific marsupial ELP and eutherian CTI share a common ancestral gene
Source: BMC Evol Biol. 2012 Jun 8;12:80. doi: 10.1186/1471-2148-12-80 (PMC3426482; doi:10.1186/1471-2148-12-80)
Supplement: Additional file 9 — Figure S6 Genomic arrangement and mVISTA plot of pairwise alignments for the bovine CTI, PTI, STI and TKDP1-5 genes. A. Arrangement and orientation of the bovine chromosome 13 CTI, PTI, STI, TKDP1 TKDP2, TKDP3, TKDP4 and TKDP5 genes. B. (i-viii) Homology between the CTI PTI, STI and TKDP1-5 genes as determined by mVISTA pairwise sequence alignment. Grey horizontal arrows indicate genes, coding exons are indicated by blue boxes and UTRs of the gene as light green rectangles. The right axis indicates the percentage identity for each pairwise comparison within a 100 bp window, ranging from 10% to 100%. Regions sharing greater than 25% identity are shaded and the black horizontal line indicates 70% identity. The horizontal axis indicates the size of the reference sequence used for each comparison: (i) Bovine CTI, (ii)PTI, (iii)STI, (iv)TKDP1, (v)TKDP2, (vi)TKDP3, (vii)TKDP4, and (viii)TKDP5. The CTI Kunitz domain was most similar to that of PTI, STI, and TKDP3, whilst PTI and STI homology was greatest within the TKDP gene family. [file 1471-2148-12-80-S9.pdf]

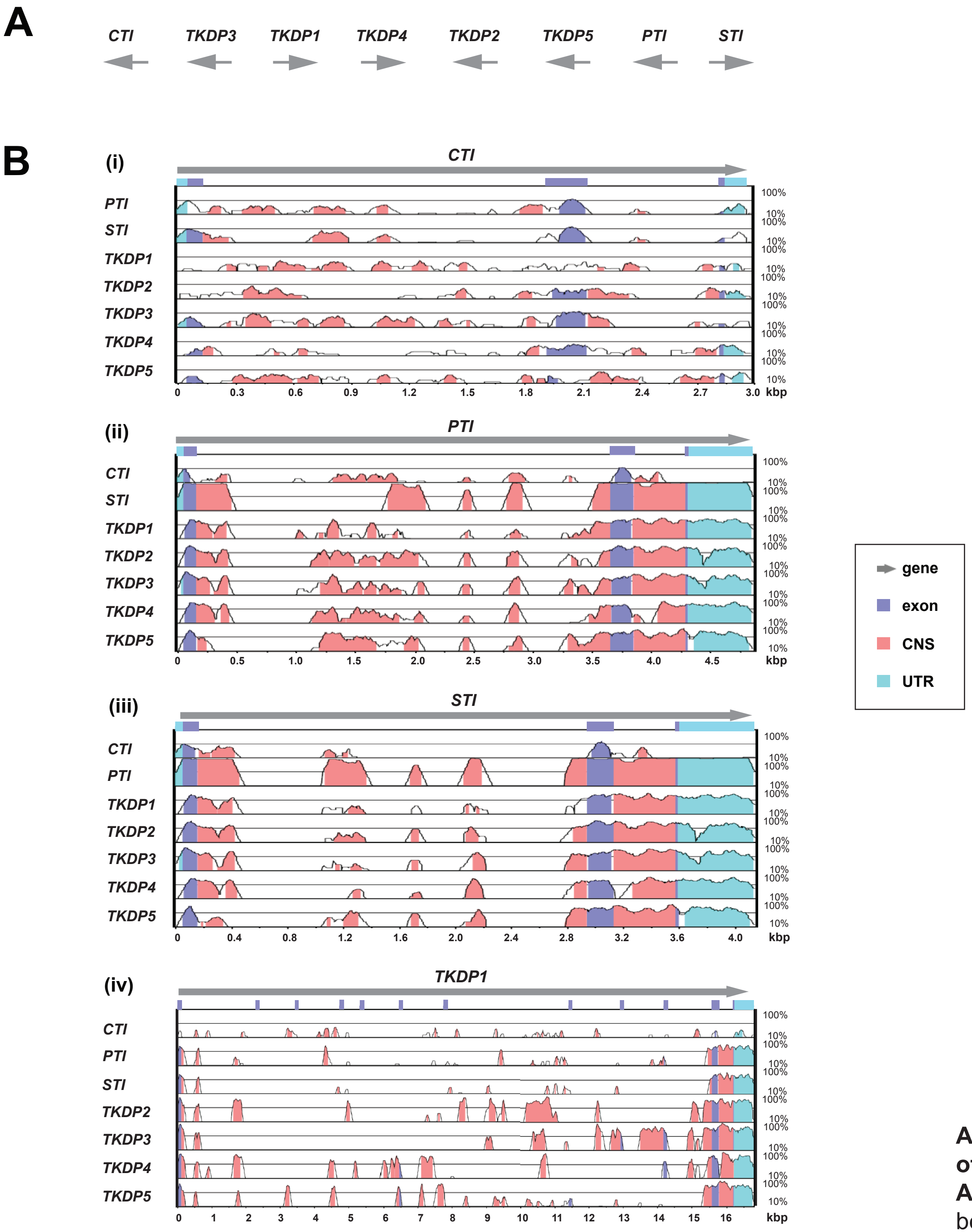

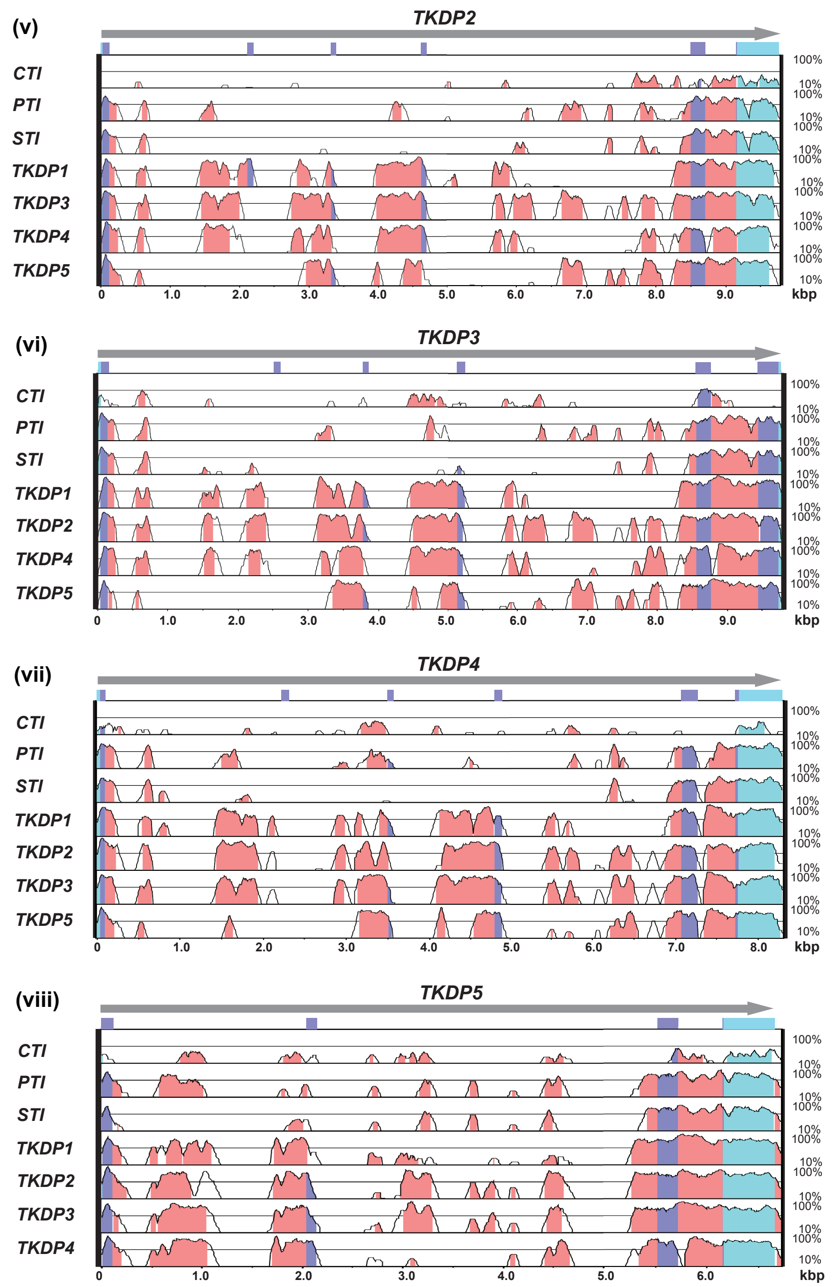

Additional file 9 - Figure S6. Genomic arrangement and mVISTA plots of pairwise alignments for the bovine *CTI*, *PTI*, *STI* and *TKDP1-5* genes
A. Arrangement and orientation and **B.** mVISTA plots of comparisons between the bovine *CTI*, *PTI*, *STI* and *TKDP1-5* genes.
