# Supplementary material for: The mammary gland-specific marsupial ELP and eutherian CTI share a common ancestral gene
Source: BMC Evol Biol. 2012 Jun 8;12:80. doi: 10.1186/1471-2148-12-80 (PMC3426482; doi:10.1186/1471-2148-12-80)
Supplement: Additional file 10 — Figure S7 Transposable elements located within the bovine CTIPTISTI and TKDP1-5 genes. Conserved transposable elements within the CTIPTISTITKDP1TKDP2TKDP3TKDP4 and TKDP5 genes (translation start to polyadenylation site, inclusive) were identified using CENSOR [66,108]. The TKDP N-domain-encoding exons located between exon 1 (signal- and pro-peptide) and the Kunitz domain-encoding exon (light blue rectangle) were most likely to have arisen due to the “exonisation” of an intron [50]. Their evolutionary history including phylogenetic and dN/dS analysis is discussed in detail [50,51,63]. The second exon of TKDP5 (A) which is adjacent to a MER21 element may be the ancestral exon of the unique 3-exon N-domains. The ancestral TKDP5 gene was probably then duplicated to produce either the ancestral TKDP4TKDP3 or TKDP2 gene. Within this copied gene, the retroelement (and the exonised intron) was most likely duplicated a further two times (B) and (C), producing a tripartite N-domain of 3 exons: C, B and A (yellow bar 1). This gene subsequently underwent 3 rounds of duplication, resulting in four genes with one N-domain, i.e. three exons which encode the N-domain. Three of these genes, TKDP4TKDP3 and TKDP2, retained the original N-domain. However, for the fourth (TKDP1) the tripartite N-domain was replicated twice (yellow bar 2 and yellow bar 3). The horizontal axis indicates the relative sizes of the regions compared, with all genes transcribed from left to right. Exons are indicated by red rectangles, with the exception of the Kunitz domain-encoding exon which is shown as a blue rectangle. Coloured rectangles indicate the different retroelement classes: Transposable elements: DNA transposon (maroon), LTR (long terminal repeat) retrotransposons (brown), Endogenous retrovirus (orange), Non-LTR retrotransposons (blue), interspersed repeat (black) and simple repeat (green). White space indicates the absence of transposable elements. Coloured lines link elements conserved between [file 1471-2148-12-80-S10.pdf]

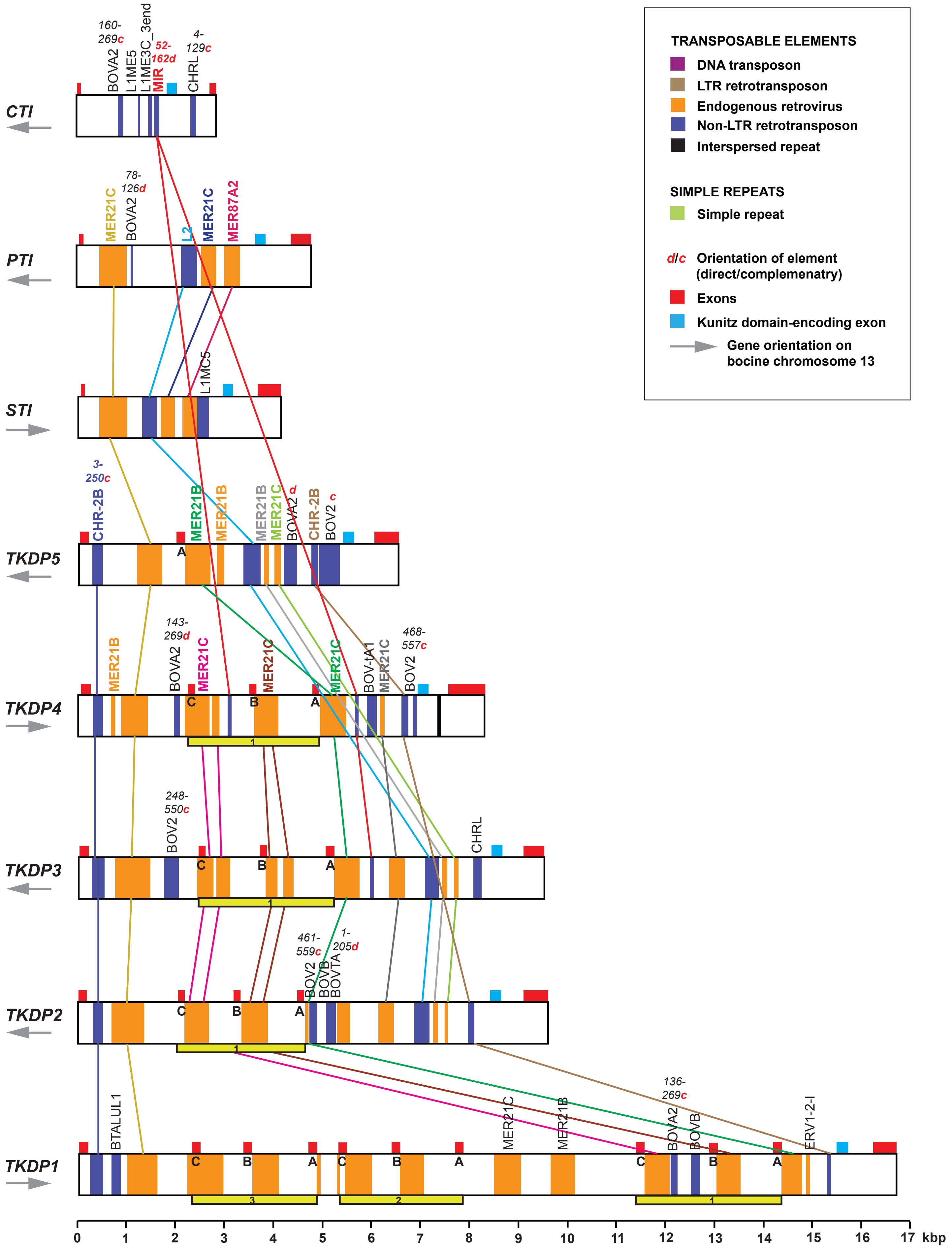

Additional file 10 - Figure S7. Transposable elements located within the bovine *CTI*, *PTI*, *STI* and *TKDP1-5* genes

A. Conserved repeat elements within the *CTI*, *PTI*, *STI* and *TKDP1-5* genes. **B.** Identity, location and orientation of transposable elements within the *CTI*, *PTI*, *STI* and *TKDP1-5* genes as determined using CENSOR.
