# Supplementary material for: The mammary gland-specific marsupial ELP and eutherian CTI share a common ancestral gene
Source: BMC Evol Biol. 2012 Jun 8;12:80. doi: 10.1186/1471-2148-12-80 (PMC3426482; doi:10.1186/1471-2148-12-80)
Supplement: Additional file 11 — Table S4 Location, identity and orientation of transposable elements within the bovine CTIPTISTI and TKDP1-5 genes. CENSOR [66,108] output tables showing the predicted identity, location and orientation of retroelement fragments within the bovine CTIPTISTITKDP1TKDP2TKDP3TKDP4 and TKDP5 genes. [file 1471-2148-12-80-S11.pdf]

Additional file 11 - Table S4. Location, identity and orientation of transposable elements within the bovine *CTI*, *PTI*, *STI* and *TKDP1-5* genes

CENSOR output tables showing the predicted identity, location and orientation of retroelement fragments within the bovine *CTI*, *PTI*, *STI*, *TKDP1*, *TKDP2*, *TKDP3*, *TKDP4* and *TKDP5* genes.

CTI

From	To	Name	From	To	Class	Dir
839	948	BOVA2	160	269	NonLTR/SINE	c
1266	1304	L1ME5	359	397	NonLTR/L1	c
1484	1566	L1ME3C_3end	426	505	NonLTR/L1	c
1612	1725	MIRb	52	162	NonLTR/SINE/SINE2	d
2388	2514	CHRL	4	129	NonLTR/SINE	d

PTI

From	To	Name	From	To	Class	Dir
400	993	MER21C	115	747	ERV/ERV3	d
1310	1624	L2	2698	3022	NonLTR/CR1	c
1705	2005	MER21C	117	403	ERV/ERV3	c
2171	2475	MER87A2_BT	500	811	ERV/ERV1	d
2484	2736	L1MC5	1859	2138	NonLTR/L1	c

STI

From	To	Name	From	To	Class	Dir
400	943	MER21C	115	690	ERV/ERV3	d
1026	1072	BOVA2	78	126	NonLTR/SINE	d
2028	2344	L2	2698	3022	NonLTR/CR1	c
2427	2721	MER21C	117	403	ERV/ERV3	c
2887	3191	MER87A2_BT	500	811	ERV/ERV1	d

TKDP1

From	To	Name	From	To	Class	Dir
221	345	CHR-2B	185	318	NonLTR/SINE	c
347	485	CHR-2B	5	144	NonLTR/SINE	c
656	847	BTALUL1	1	193	NonLTR/SINE/SINE2	c
971	1584	MER21C	238	909	ERV/ERV3	d
2200	2926	MER21C	105	911	ERV/ERV3	d
3518	4053	MER21C	316	903	ERV/ERV3	d
4833	4908	MER21C	320	402	ERV/ERV3	d
5241	5298	MER21C	115	174	ERV/ERV3	d
5410	5948	MER21C	317	909	ERV/ERV3	d
6522	7010	MER21C	280	815	ERV/ERV3	d
8437	8980	MER21C	317	909	ERV/ERV3	d
9587	10083	MER21B	171	715	ERV/ERV3	d
11498	12005	MER21C	317	848	ERV/ERV3	d
12033	12168	BOVA2	136	269	NonLTR/SINE	c
12438	12621	BovB	3664	3847	NonLTR/RTE	c
12965	13449	MER21B	171	715	ERV/ERV3	d
14282	14704	MER21C	316	786	ERV/ERV3	d
14783	14861	ERV1-2-I_BT	2917	2995	ERV/ERV1	c
15212	15289	CHR-2B	1	78	NonLTR/SINE	c

TKDP2

From	To	Name	From	To	Class	Dir
291	492	CHR-2B	3	245	NonLTR/SINE	c
674	1348	MER21C	115	848	ERV/ERV3	d
2191	2692	MER21C	316	864	ERV/ERV3	d
3371	3917	MER21C	306	877	ERV/ERV3	d
4698	4766	MER21C	318	387	ERV/ERV3	d
4789	4888	BOV2	461	559	NonLTR/SINE	c
4889	4943	BovB	3095	3149	NonLTR/RTE	c
5127	5328	BOVTA	1	205	NonLTR/SINE	d
5358	5629	MER21C	596	904	ERV/ERV3	d
6214	6536	MER21C	106	450	ERV/ERV3	d
6957	7276	L2	2713	3032	NonLTR/CR1	c
7353	7452	MER21B	207	308	ERV/ERV3	c
7594	7661	MER21C_BT	49	116	ERV/ERV3	c
8071	8208	CHR-2B	1	133	NonLTR/SINE	c

TKDP3

From	To	Name	From	To	Class	Dir
225	352	CHR-2B	174	315	NonLTR/SINE	c
359	482	CHR-2B	5	129	NonLTR/SINE	c
708	1433	MER21C	125	909	ERV/ERV3	d
1717	2016	BOV2	248	550	NonLTR/SINE	c
2390	2727	MER21C	103	473	ERV/ERV3	d
2788	3066	MER21B	466	763	ERV/ERV3	d
3805	4048	MER21C	285	542	ERV/ERV3	d
4168	4374	MER21C	596	825	ERV/ERV3	d
5213	5735	MER21C	316	863	ERV/ERV3	d
5947	6030	MIRc	62	162	NonLTR/SINE/SINE2	d
6352	6670	MER21C	106	445	ERV/ERV3	d
7083	7365	L2	2747	3027	NonLTR/CR1	c
7428	7539	MER21B	209	321	ERV/ERV3	c
7681	7772	MER21C_BT	23	116	ERV/ERV3	c
8081	8249	CHRL	1	168	NonLTR/SINE	c

TKDP4

From	To	Name	From	To	Class	Dir
291	497	CHR-2B	3	247	NonLTR/SINE	c
660	744	MER21C	1	81	ERV/ERV3	d
871	1411	MER21B	223	801	ERV/ERV3	d
1935	2061	BOVA2	143	269	NonLTR/SINE	d
2155	2654	MER21C	115	656	ERV/ERV3	d
2705	2858	MER21C	730	909	ERV/ERV3	d
3023	3099	MIRc	62	152	NonLTR/SINE/SINE2	d
3551	4046	MER21C	271	816	ERV/ERV3	d
4889	5420	MER21C	316	905	ERV/ERV3	d
5599	5670	MIRc	62	151	NonLTR/SINE/SINE2	d
5844	6041	Bov-tA1	4	211	NonLTR/SINE/SINE2	d
6105	6203	MER21C_BT	12	116	ERV/ERV3	c
6547	6682	CHR-2B	3	137	NonLTR/SINE	c
6772	6856	BOV2	468	557	NonLTR/SINE	d
7271	7339	LSU-rRNA_Hsa	2163	2234	Pseudogene/rRNA	c

TKDP5

From	To	Name	From	To	Class	Dir
268	476	CHR-2B	3	250	NonLTR/SINE	c
1169	1680	MER21C	316	864	ERV/ERV3	d
2142	2648	MER21B	213	755	ERV/ERV3	d
2785	2931	MER21B	213	355	ERV/ERV3	d
3318	3660	L2	2699	3046	NonLTR/CR1	c
3726	3832	MER21B	208	315	ERV/ERV3	c
3941	4069	MER21C	1	120	ERV/ERV3	c
4126	4394	BOVA2	1	269	NonLTR/SINE	d
4689	4823	CHR-2B	2	133	NonLTR/SINE	c
4843	5254	BOV2	150	560	NonLTR/SINE	c
